# Enhanced Efficacy of Pomegranate Peel Extract via Double Nano-Emulsion Delivery in Laying Hens: Impact on Performance, Immunity, Antioxidant Status, and *Salmonella Typhimurium* Resistance

**DOI:** 10.3390/vetsci13080775

**Published:** 2026-08-02

**Authors:** Hanan S. Al-Khalaifah, Asmaa T. Y. Kishawy, Rania M. S. El-Malt, Wessam Youssef, Walaa A. Habib, Dalia W. A. H. Elged, Wafaa M. Gad, Eman A. Elalfy, Mohammed E. E. Sayed Ahmed, Hebatullah M. Abouelfadl, Nanies S. E. Salim, Marwa M. Fathi, Doaa Ibrahim

**Affiliations:** 1 Environment and Life Sciences Research Center, Kuwait Institute for Scientific Research, P.O. Box 24885, Safat 13109, Kuwait; 2 Department of Nutrition and Clinical Nutrition, Faculty of Veterinary Medicine, Zagazig University, Zagazig 44511, Egypt; 3 Department of Microbiology, Animal Health Research Institute (AHRI), Zagazig Branch, Agriculture Research Center (ARC), Zagazig 44516, Egypt; 4 Department of Biotechnology, Animal Health Research Institute (AHRI), Agriculture Research Center (ARC), Giza 12618, Egypt; 5 Department of Biochemistry, Animal Health Research Institute (AHRI), Zagazig Branch, Agriculture Research Center (ARC), Zagazig 44516, Egypt; 6 Department of Toxicology and Biochemistry, Animal Health Research Institute (AHRI), Zagazig Branch, Agricultural Research Centre (ARC), Zagazig 44516, Egypt; 7 Department of Bacteriology, Animal Health Research Institute (AHRI), Mansoura Branch, Agriculture Research Center (ARC), Mansoura 35511, Egypt; 8 Department of Pharmacology and Biochemistry, Animal Health Research Institute (AHRI), Mansoura Branch, Agriculture Research Center (ARC), Mansoura 35511, Egypt; 9 Department of Bacteriology, Immunology and Mycology, Animal Health Research Institute (AHRI), Mansoura Branch, Agriculture Research Center (ARC), Mansoura35511, Egypt; 10 Department of Pharmacology, Animal Health Research Institute (AHRI), Agricultural Research Center (ARC), Giza 12618, Egypt; 11 Biochemistry Department, Zagazig Branch, Animal Health Research Institute (AHRI), Agricultural Research Centre (ARC), Zagazig 44516, Egypt; 12 Serology Unit, Animal Health Research Institute (AHRI), Agriculture Research Center (ARC), Dokki, P.O. Box 246, Giza 12618, Egypt

**Keywords:** pomegranate peel extract nano-emulsion, antioxidant, laying performance, immunostimulant, anti-inflammatory, anti-virulence, *Salmonella Typhimurium*

## Abstract

Multidrug-resistant (MDR) *Salmonella Typhimurium*, largely spread through poultry products, increasingly resists antibiotic treatment. We evaluated dietary pomegranate peel extract-loaded nano-emulsions (PomNEs) on performance, immunity, antioxidant capacity, and *S. Typhimurium* resistance in laying hens. A total of 250 15-week-old Hy-Line Brown hens received a basal diet or diets supplemented with PomNEs at 0.3, 0.6, or 1.2 g/kg, and then were challenged with *S. Typhimurium* at 34 weeks. Hens fed PomNEs, especially PomNEs1.2, showed improved egg production and feed efficiency before challenge and restored normal output afterward. Supplementation reduced *Salmonella* colonization in the ovaries, liver, and eggs, with considerable reduction in eggs and ovaries at the highest dose by ten weeks, and down-regulated bacterial virulence genes (*hilA*, *invA*). Immune function improved through higher phagocytic activity, intracellular killing, and lysozyme levels, lower nitric oxide, up-regulated *IgA* and β-defensins (*AvBD6*, *AvBD12*), and suppressed pro-inflammatory cytokines and chemokines. Intestinal and ovarian redox balance improved via reduced *COX2* and elevated *GPX-1*, *HO-1*, *NQO1*, *SOD-1*, and *CAT* expression. These benefits support PomNEs as promising natural feed additives to enhance laying performance, antioxidant defense, and immunity against salmonellosis, offering a sustainable strategy for supporting disease control in poultry production.

## Simple Summary

Drug-resistant *Salmonella Typhimurium* is a major concern in poultry because it can be transmitted to humans through contaminated eggs and meat. This study evaluated the effects of a double nano-emulsion of pomegranate peel extract, a natural plant-derived product rich in antioxidants, as a dietary supplement for laying hens. A total of 250 Hy-Line Brown hens were fed a basal diet or diets supplemented with the extract at 0.3, 0.6, or 1.2 g/kg before experimental challenge with *Salmonella Typhimurium*. Supplementation with the nano-encapsulated extract was associated with improved laying performance, better feed efficiency, and faster recovery following infection, particularly at the highest dose. Higher supplementation levels were also associated with lower *Salmonella* counts in the ovaries, liver, and eggs, together with changes in immune, inflammatory, bacterial virulence, and antioxidant-related biomarkers. These findings suggest that nano-encapsulated pomegranate peel extract has potential as a natural feed additive to support laying hen health and productivity while contributing to improved control over *Salmonella* contamination in poultry production.

## 1. Introduction

Improvements in birds’ productivity, feed conversion ratios, and the control of gastrointestinal microbial illnesses have led to an elevation in consumer demand for poultry-based food, especially in developing countries. *Salmonella* is among the most common bacteria impacting poultry diseases [1]. *Salmonella enterica* serotype Typhimurium is a common enteric bacterial pathogen that is associated with human food-borne diseases due to consumption of contaminated poultry products, such as meat and eggs, and is most commonly identified in infected laying hens and asymptomatic poultry flocks, causing considerable economic loss to the poultry industry [2,3]. *Salmonella* egg contamination may occur through vertical transmission from the colonized reproductive organs (oviduct and ovary) prior to shell formation, and horizontal transmission by the external contamination of the eggshell from the feces of infected laying hens [4]. *Salmonella Typhimurium* has demonstrated the ability to endure and proliferate within bird macrophages, which is critical for its virulence [5]. *Salmonella* species can express numerous virulence-related proteins, especially invasion protein regulator, which is encoded by the *hilA* gene that regulates the type III secretion system, facilitating the expression of invasion genes [6]. Furthermore, one of the most important virulence factors utilized as a biomarker for *Salmonella* species is invasin protein A (encoded by the *invA* gene), which is present in the outer membrane of the bacteria and facilitates entry into the enteric epithelium, hence starting the infection [5]. Moreover, the initiation of intestinal inflammatory reactions is facilitated by a unique set of cytokines generated in response to Salmonella infection [3,7]. The widespread usage of antibiotics to manage microbial infections, such as salmonellosis, and as promoting agents in poultry production has resulted in the emergence of multidrug-resistant (MDR) bacteria in both animals and humans, reducing the effectiveness of therapies and increasing the risk of mortality [8,9,10]. Consequently, researchers are investigating innovative substitutes for antimicrobials, such as phytogenics [11]. *Salmonella* spp. have developed resistance to several antimicrobials, resulting in MDR forms that have multiplied in both developing and developed nations [12]. Furthermore, *Salmonella* spp. can act as reservoirs and vectors for the spread of antimicrobial resistance in many contexts, thus posing a considerable public health threat [13]. Salmonellosis in chickens, especially in young chicks, is correlated with significant fatality and morbidity, poor growth, and reduced feed efficiency; concurrently, infected adult birds may serve as asymptomatic carriers, promoting vertical transmission via contaminated eggs, leading to considerable economic losses in the avian sector [13,14]. Hence, it is crucial to adopt effective approaches for mitigating and minimizing salmonellosis in poultry production. Diverse techniques, including phytogenics, may be employed in conjunction with biosecurity policies inside farms, as diverse mechanisms of action can produce beneficial outcomes throughout the egg and meat production cycles, thereby improving the safety of eggs and meats [3]. To preserve gut health and diminish the reliance on antimicrobial agents for intestinal infections, alternatives are necessary in both animal and human healthcare, along with modern consumer demand for non-medicated, natural products [15]. Phytogenics, such as flavonoids, are produced by plants through both kinds of metabolism and serve as natural alternatives to antibiotics [16]. Recent research confirms that phytogenics, such as polyphenols, exhibit diverse pharmaceutical properties because of their antioxidant, immunostimulant, antimicrobial, anti-inflammatory, and anti-virulence activities [17]. Pomegranate and its derivatives constitute a substantial source of several phenolic compounds, affirming its status as a powerful medicinal herb [18]. Pomegranate peel extract (PPE) has demonstrated antimicrobial, antioxidant, immunostimulant, growth-enhancing, and anti-stress properties in chickens across many investigations [19,20]. Despite these advantages, phenolic substances, such as PPE, are vulnerable to several environmental factors, including pH, light, and oxygen. Encapsulation serves as an effective strategy to guard them in these situations and promote their effectiveness [21]. Nano-encapsulation of sensitive bioactive substances, such as PPE, preserves and enhances their physical and biological characteristics by shielding them from chemical, enzymatic, and environmental alterations [21]. This process also facilitates their controlled dispersal and alleviates the drawbacks associated with the unpleasant flavor of polyphenols, effectively masking their astringent and unpleasant taste, which limits their immediate use [22]. Notwithstanding these advancements, evidence for the in vivo effectiveness of PPE-loaded nano-emulsions (PomNEs) in laying hens is limited, especially under experimental infection circumstances. According to available evidence, a few studies have explored the prophylactic or therapeutic impacts of PomNEs in laying hens experimentally challenged with *S. Typhimurium*, with its influence on host physiological reactions and bacterial virulence. Building on this rationale, we formulated the primary hypothesis that dietary supplementation with pomegranate peel extract-loaded double nano-emulsions (PomNEs) would alleviate the detrimental effects of MDR *S.* Typhimurium infection in laying hens through a coordinated host- and pathogen-directed action, namely, by enhancing systemic and intestinal immune competence and endogenous antioxidant defenses and reducing bacterial colonization of the reproductive organs and eggs, thereby preserving laying performance. We additionally hypothesized that nano-encapsulation, by improving the stability and bioavailability of pomegranate polyphenols, would enable these protective effects to be achieved at comparatively low dietary inclusion levels. To test these hypotheses, the present research sought to investigate, in laying hens, the effectiveness of pomegranate peel extract double-loaded nano-emulsions on serum and blood immune indicators, and the mRNA transcript levels of genes encoding antioxidant biomarkers, immune response, and inflammatory regulators, as well as the regulation of *S. Typhimurium* virulence and colonization in hens experimentally infected with MDR *S. Typhimurium*.

## 2. Materials and Methods

### 2.1. Ethical Approval

The research experiment was executed in compliance with the ARRIVE instructions for the husbandry and management of laying hens, which adhered to the standards set by the Animal Care and Use Committee of the Faculty of Veterinary Medicine at Zagazig University, Egypt (ZU-IACUC/2/F/62/2024).

### 2.2. Preparation and Characterization of Pomegranate Peel Extract-Loaded Nano-Emulsions

**Figure 1:**
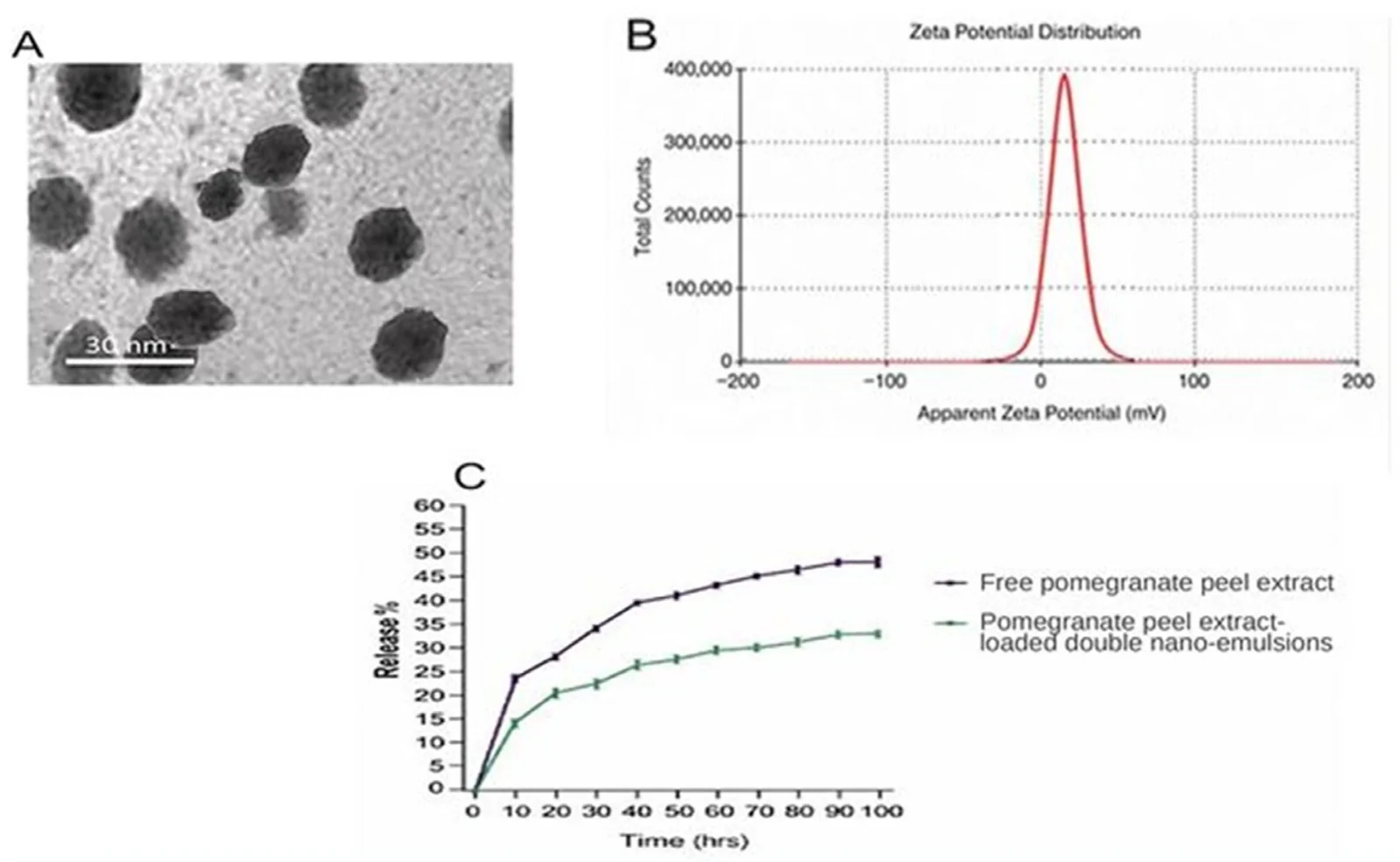
(**A**) The transmission electron microscopy (TEM) particle size was 157 nm. (**B**) Zeta potential of +25 Mv of pomegranate peel extract-loaded nano-emulsions. (**C**) Release percentage of pomegranate peel extract in free and nano-loaded forms 0.25 dpi.

Pomegranate peel extract was prepared using the methodology developed by Oliveira, Castro [23]. Peels from pomegranates were carefully ground, dried at 50 °C, and then extracted by 70% ethyl alcohol. The blend was then purified after being incubated at an ambient temperature for 24 h in the dark. After being dried out, the extracted material was lyophilized by freezing for 24 h at −70 °C. It was then stored at −20 °C in a container that was shielded from light. The polyphenolic fractions of the generated PPEs were measured using a high-performance liquid chromatography (HPLC) technique (detailed quantification of analysis in Table S1). The two stages of pomegranate nano-loading were performed as follows. First, an oil-in-water emulsion consisting of nine parts water to one part oil was used to create the pomegranate peel extract-loaded nano-emulsions (PomNEs). A hotplate with a magnetic stirrer (MS300HS; Mtops, Yangju-City, Republic of Korea) was used to mix 10 mL of sunflower oil with 1 g of lecithin, which served as a surfactant, for 1.5 h at 70 °C in order to prepare the oil phase. Ten grams of the extract were progressively added under continuous stirring until the lecithin had completely dissolved. After that, while stirring, the mixture was gradually added to 90 mL of water, the aqueous phase. After 20 more minutes of mixing, the emulsion was homogenized for 15 min at 25 MHz using a probe sonicator (Ultrasonic Egypt, Cairo, Egypt). For later use, the PomNEs were then kept in vials at −20 °C [24]. The second phase of nano-loading consisted of a 0.1 M NaCl solution, in which Tween 20 and carboxymethyle cellulose (Sigma-Aldrich St. Louis, MO, USA) (0.5% *w*/*w*) were dissolved. The final two emulsions were first homogenized using a high-speed homogenizer at 6000 rpm for 4 min, followed by sonication for 1.5 min at a frequency of 24 kHz and 30% amplitude. The encapsulation efficiency was determined according to [25] as follows. First, the free extract was separated from the encapsulated one by a Centrifugal Ultrafiltration Amicon^®^ Ultra centrifugal filter unit (Merck Millipore/Millipore Sigma-Aldrich, St. Louis, MO, USA). Then, the bioactive content was quantified by HPLC, and finally, the encapsulation efficiency (EE) was calculated using the following equation: EE% = (total extract added − free extract detected)/total extract added × 100, which yielded 96.15%. Transmission electron microscopy (TEM) (JSM-6360 LA; JEOL, Tokyo, Japan) was used to assess the morphology and size of the PN particles. The single-layer encapsulation size was 48 nm and the overall double-encapsulated particle size was 157 nm. The hydrodynamic diameter and polydispersity index were characterized by dynamic light scattering (DLS) using a Zetasizer Nano ZS (Malvern Instruments, Malvern, UK) at 25 °C and were calculated as 157 nm and a PDI of 0.25 respectively, while the surface charge was assessed through zeta potential measurements performed on the same instrument, which was +25 mV (Figure 1).

### 2.3. Hens, Experimental Design, and Diet Formulation

**Figure 2:**
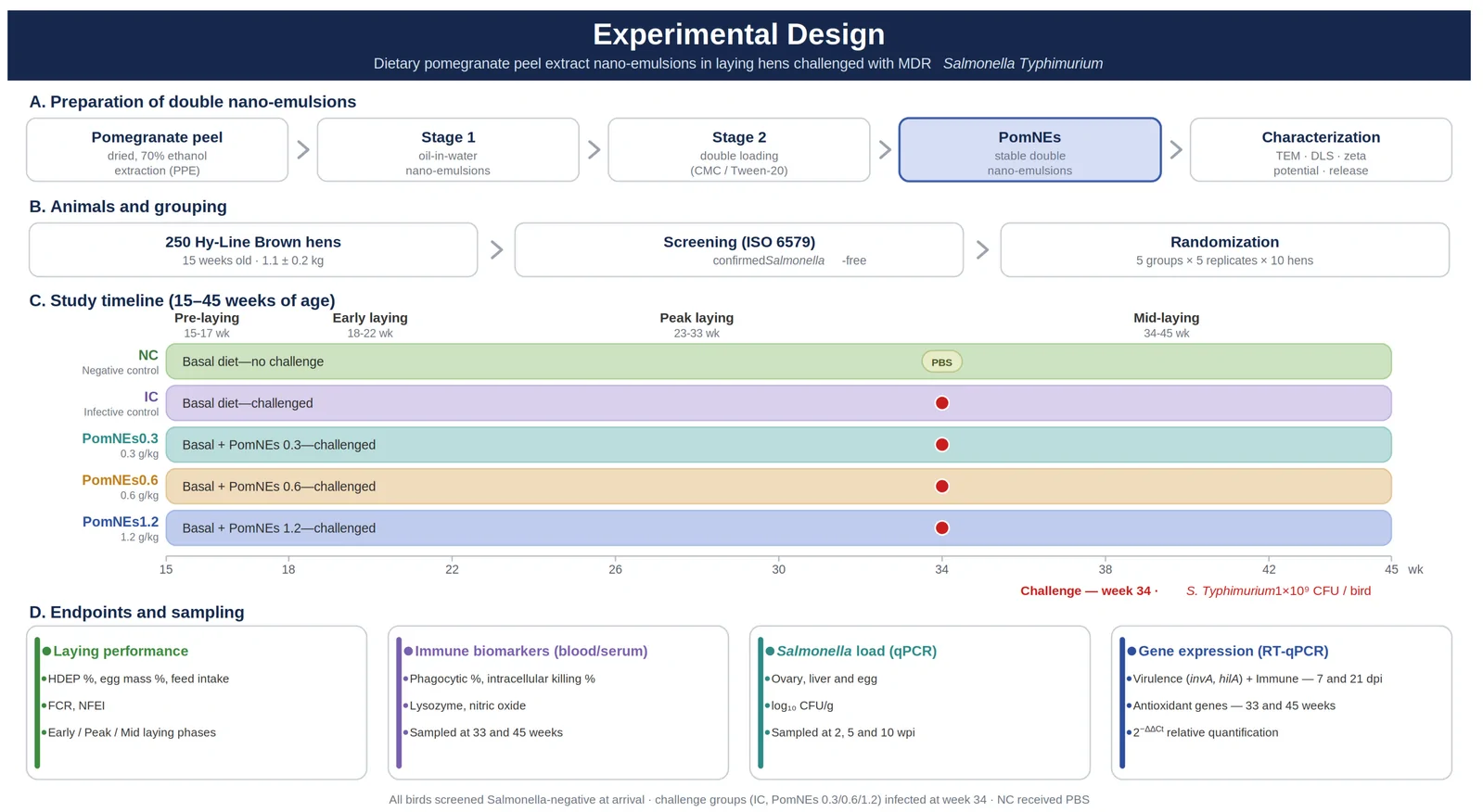
Diagram explaining the experimental design.

A total of 250 15-week-old Hy-Line Brown laying hens with a primary body weight of 1.1 ± 0.2 kg were bought commercially from nearby producers. In compliance with International Organization for Standardization (ISO) 6579 criteria [26], the hens underwent a bacteriological investigation consisting of cloaca swabs for fecal specimens upon arrival to verify the absence of *Salmonella* spp. [27]. After being weighed, the chickens were randomly assigned to five equal experimental groups (randomization was done by weighting all hens individually and assigning each a unique identification number). Birds were then allocated to experimental groups using a random sequence while ensuring a similar distribution of initial body weights across groups. Statistical analysis confirmed that there were no significant differences in mean initial body weight among the groups (*p* > 0.05), (each cage or pen represents a replicate). Birds were kept inside a battery enclosure system (height: 45 cm, width: 120 cm, and depth: 50 cm) with a photoperiod of 16 h per day, relative humidity between 60 and 70%, and relative temperature between 25 and 27 °C. Throughout the day, they had unlimited access to food and water. Throughout the experimental trial, two pelleted rations were provided: a pre-lay diet fed before the onset of laying (15 to 17 weeks) and a laying diet fed during egg production (18 to 45 weeks) (Table 1). The feed materials were thoroughly mixed and then pelletized at a pressure of 1.5 kg/cm^2^ in a steam pellet mill (Koppers Junior C40, Koppers Company, Pittsburgh, PA, USA) to a size of around 2.5 mm. Each diet was formed to meet the dietary demands of Hy-Line Brown laying hens in compliance with their nutritional specification catalog. The five experimental groups were constituted as follows: a negative control (NC) group, where hens were administered a basic diet devoid of additions, and an infected control (IC) group, which consisted of hens that received a basic diet devoid of additions and were challenged with *S. Typhimurium* (1 × 106 CFU/bird) at 34 weeks of age. Groups PomNEs0.3, PomNEs0.6, and PomNEs1.2 were fed a basal diet fortified with pomegranate peel extract-loaded nano-emulsions (PomNEs) at dosages of 0.3, 0.6, and 1.2 g/kg of feed, respectively, within ranges of previous studies [28,29,30], and subsequently challenged with *S. Typhimurium* at a dose of 1 × 106 CFU per bird at 34 weeks of age (Figure 2). The study period spanned 30 weeks, commencing with 15-week-old hens and concluding when the hens were 45 weeks old. Table 1 presents the chemical makeup of the base diet and the levels of feed ingredients. The evaluation of the feed constituents was conducted in accordance with the typical protocols of the AOAC [31].

### 2.4. Salmonella Typhimurium Challenge Model

The current challenge trial used a field-isolated MDR *S. Typhimurium* strain that was identified phenotypically utilizing traditional bacteriological techniques from salmonellosis cases in a local broiler farm in Sharkia Governorate, Egypt. Briefly, the strain was grown on Rappaport-Vassiliadis broth (Oxoid, Basingstoke, UK) and incubated at 42 °C for 24 h. Thereafter, a loopful of each broth was inoculated onto Salmonella-Shigella agar, xylose lysine deoxycholate agar, and MacConkey’s agar plates (Oxoid, Basingstoke, UK), and the resultant colonies underwent Gram staining, followed by microscopic analysis. Additional identification was conducted using biochemical techniques, encompassing responses on triple sugar iron agar and lysine iron agar, indole synthesis in tryptone broth, carbon assimilation in Simmon’s citrate agar, and urea hydrolysis in Christensen’s urea agar. Standard slide and tube agglutination tests employing commercial polyvalent and monovalent O and H antisera (Denka-Seiken, Tokyo, Japan) were utilized to serotype typical Salmonella isolates and identify Salmonella serovars. The strain was validated via PCR detection of the *invA* gene, a *Salmonella*-specific gene, in accordance with a previously established method [33]. The strain’s antibiotic susceptibility profile was assessed according to the Clinical and Laboratory Standards Institute (CLSI) guidelines (CLSI, 2023) [34]. The strain demonstrated resistance to many microbes, thereby being classified as multidrug-resistant (MDR) (Table S2). Additionally, PCR analysis validated its pathogenicity by detecting the *invA* and *hilA* genes, utilizing previously described primers and PCR cycle specifications [33]. The challenge strain was cultured in brain heart infusion (BHI) broth (Oxoid, Basingstoke, UK), passed two times in healthy birds to augment its virulence, and then re-isolated from killed birds for application in the trial. All birds were subjected to bacteriological analysis to verify their absence of *S. Typhimurium* before commencing the challenge experiment, in compliance with International Organization for Standardization (ISO) 6579 criteria (ISO, 2017) [26]. To produce the challenge bacterial inoculum for oral gavage of birds, the bacterial suspension was serially diluted in BHI broth until the final viable cell titer reached 1 × 10^6^ CFU/mL. At 34 weeks, the experimental birds (8 birds/replicate) were orally infected with *S. Typhimurium* (1 × 10^6^ CFU/bird) using a syringe fitted with flexible tubing, while the negative control group received a PBS gavage. Throughout the trial, the infected hens were kept under careful observation. Using standard bacteriological procedures and PCR assays as outlined by [33], the *S. Typhimurium* challenge was verified by re-isolating the challenged strain from liver, ovaries, and egg samples of the challenged hens. From the moment of the challenge to the end of the experiment, clinical symptoms, fatalities, and gross lesions were documented.

### 2.5. Performance of Laying Hens

Every performance metric was carried out in accordance with the Hy-line management catalog (Hy-Line International, 2025). Each replicate had its eggs collected three times a day at 8:00 am, 1:00 pm, and 6:00 pm. We noted the weight and quantity of eggs as well as the daily feed consumption for each replicate by dividing pen feed consumption by the number of birds to compute the hen-day egg production percentage (HDEP), average egg mass for the period percentage, total feed intake (FI), feed conversion ratio (FCR) and net feed efficiency index (NFEI), as formerly described by [35].

### 2.6. Collection of Samples

At the conclusion of the peak laying phase (33 weeks) and at the conclusion of the mid-laying period (45 weeks), blood specimens were drawn under an aseptic procedure from the birds’ wing vein (five specimens per group). The specimens were split into two equal portions, with the first portion containing EDTA (Sigma-Aldrich, St. Louis, MO, USA) as an anticoagulant for assessing phagocytosis and the other portion being obtained without EDTA for serum separation, which was utilized in immune evaluations. At 33 weeks (before challenge, five birds/group) and 45 weeks (after challenge, five birds/group), intestinal and ovarian tissues were collected directly on TRIzol reagent (Invitrogen Life Technologies, Carlsbad, CA, USA) as per the manufacturer’s guidelines and subsequently stored at −80 °C for downstream gene expression analysis of antioxidant-related genes by quantitative reverse transcription polymerase chain reaction (RT-qPCR) assay. At 7 and 21 days post-infection (dpi), intestinal tissues, after being collected on TRIzol (Sigma-Aldrich, St. Louis, MO, USA) reagent, were employed for measuring the transcript levels of anti-inflammatory and *S. Typhimurium* virulence genes by RT-qPCR assay. At 2, 5, and 10 weeks post-infection (wpi), ovary, liver, and egg samples were aseptically gathered and employed for subsequent measurement of *S. Typhimurium* via quantitative real-time PCR (qPCR) assay. All investigators responsible for laboratory analyses were blinded to treatment allocation.

### 2.7. Blood and Serum Immunological Analysis

At 33 weeks (prior to the challenge) and at 45 weeks (following the challenge), blood and serum samples (N = 5 per group) were used for determining the phagocytic percentage, intracellular killing capacity (ICK), lysozyme (LYZ), and nitric oxide (NO) levels. Commercial diagnostic kits (Nanjing Jiancheng Bioengineering Institute, Nanjing, China) were utilized to measure serum concentrations of NO and LYZ as per the manufacturer’s directions. A phagocytosis experiment was carried out using the approach outlined by [36] to calculate the phagocytic percentage and intracellular killing capacity (ICK).

### 2.8. Salmonella Typhimurium DNA Copy Counting Using a Quantitative Real-Time PCR Method

The egg, ovarian, and liver tissues were used for DNA extraction at 2, 5, and 10 weeks post-infection (wpi) with *S. Typhimurium* for counting the *S. Typhimurium* load. The QIAamp DNA Stool Mini Kit (Qiagen, Hilden, Germany) was employed for DNA extraction in accordance with the manufacturer’s instructions. The Spectrostar Nano Drop 2000 spectrophotometer (Thermo Fisher Scientific Inc., Waltham, MA, USA) was utilized to spectrophotometrically assess the purity and quantities of extracted bacterial DNA. Refined DNA was put in storage at −80 °C for further qPCR analysis. The PCR amplification was conducted in triplicate using 2x QuantiTect Probe RT-PCR Master Mix (Qiagen, Hilden, Germany) and a Stratagene MX3005P RT-PCR machine (Qiagen, Hilden, Germany). The PCR primer specific to the Salmonella *invA* gene and its respective cycle characteristics were previously established [37]. The primer sequences targeting the *invA* gene specific to *S. Typhimurium* are displayed in Table 2. A standard curve was created to quantify numbers of DNA copies. To ascertain the threshold cycle (Ct) value associated with each dilution, DNA obtained from unpolluted bacterial strains were consecutively diluted ten times before being measured in a qPCR run. By interpolating the Ct values of each DNA sample into the established standard calibration curves, the concentrations of *S. Typhimurium* were determined and displayed as log10 CFU/g sample.

### 2.9. Quantitative Analysis of Gene Expression by RT-qPCR

At 33 weeks (before challenge) and 45 weeks (after challenge), ovarian and intestinal tissues were utilized to evaluate the transcript levels of antioxidant-encoding genes, including NAD (P), H dehydrogenase quinone 1 (*NQO1*), haemoxygenase-1 (*HO-1*), cyclooxygenase-2 (*COX-2*), catalase (*CAT*), glutathione peroxidase (*GPX-1*), and superoxide dismutase (*SOD-1*). At 7 and 21 dpi with *S. Typhimurium*, intestinal tissues were taken to evaluate the *S. Typhimurium* virulence genes (*invA* and *hilA*), as well as immune-related genes, including interleukins (*IL-6*, *IL-1β*), tumor necrosis factor (*TNF-α*), chemokines [C–C motif ligand 20 (*CCL20*) and stromal cell-derived factor-1 (*CXCL13*)], immunoglobulin A (*IgA*), and antimicrobial peptide-related genes [Avian β-defensin 6 and 12 (*AvBD6* and *AvBD12*)]. Total RNA was isolated using the QIAamp RNeasy Mini kit (Qiagen, Hilden, Germany), and its quantity was assessed via a Spectrostar NanoDropTM 2000 spectrophotometer (Thermo Fisher Scientific Inc., USA) at 260 nm optical density. The one-step RT-qPCR assay was validated using the Stratagene MX3005P real-time PCR with the QuantiTect SYBR Green RT-PCR kit (Qiagen, Hilden, Germany). A concluding melting curve assessment was used to confirm the specificity of each PCR amplification. Glyceraldehyde 3-phosphate dehydrogenase (*GAPDH*) was used as an endogenous (housekeeping) control for poultry genes, and RNA polymerase sigma factor (*rpoD*) was used as an endogenous (housekeeping) control for bacteria genes to normalize the transcription levels. Table 2 lists the target genes’ primer sequences [38]. The analyzed data about the relative mRNA transcript of the targeted genes under investigation were assessed using the 2^−∆∆Ct^ method [40].

### 2.10. Statistical Analysis

All data were analyzed using SPSS Statistics v. 26 (IBM Corp., Armonk, NY, USA), and graphs were generated with GraphPad Prism v. 8 (GraphPad Software, San Diego, CA, USA). Prior to analysis, the normality of the data distribution was verified across all treatment groups using the Shapiro–Wilk test, and homogeneity of variances was assessed using Levene’s test.

Single-time-point comparisons among groups—productive performance traits, blood and serum immunological biomarkers, and the relative mRNA expression of virulence, immune-related, and antioxidant-related genes were analyzed by one-way ANOVA within the general linear model (GLM) framework. For parameters assessed at more than one time point (e.g., before and after the challenge, or at 7 and 21 days post-infection), groups were compared separately at each time point. When ANOVA indicated significant differences among groups, means were separated using Tukey’s post hoc test.

The pathogen clearance kinetics *S. Typhimurium* loads quantified in the ovary, liver, and egg at 2, 5, and 10 weeks post-challenge (Figure 4; liver clearance kinetics in Figure 6A) were analyzed by repeated-measures analysis, with treatment group as the between-subjects factor and sampling time as the within-subjects (repeated) factor. Bacterial loads were expressed as log10 CFU/g.

Relative gene expression was calculated as fold change relative to the infected control group (set to 1.0) using the 2^−ΔΔCt^ method. Host (chicken) target genes were normalized to *GAPDH*, whereas the *S. Typhimurium* virulence genes (*invA* and *hilA*) were normalized to the bacterial reference gene rpoD.

A recovery index was calculated for each treated group to express the proportion of the infection-induced deficit (the gap between the infected control [IC] and negative control [NC] groups) restored toward the NC level. This normalization rescales each treatment mean onto a 0–100% interval bounded by the two control groups, analogous to the percentage-of-maximal-possible-effect approach used to express treatment responses relative to a defined reference range while accounting for differing baseline deficits [41,42]. Recovery (%) = [(treatment mean − IC mean)/(NC mean − IC mean)] × 100. For the feed conversion ratio, where lower values are favorable, the index was inverted: [(IC mean − treatment mean)/(IC mean − NC mean)] × 100. Values exceeding 100% indicate restoration beyond the uninfected control level. As a descriptive transformation of the group means, the recovery index was not itself subjected to inferential testing; instead, each estimate was compared against complete recovery (the 100% reference) using a one-sample z-test [z = (R − 100)/propagated SE].

## 3. Results

### 3.1. The Effectiveness of Pomegranate Peel Extract-Loaded Nano-Emulsions on Egg-Laying Performance

Table 3 depicts the effect of dietary inclusion of graded concentrations of PomNEs throughout various laying phases. During the early egg production phase (before challenge), HDEP % showed the most substantial elevation (*p* < 0.05) in hens offered PomNEs1.2 when compared with the negative and infected control (NC, IC) groups. During the peak of the egg production phase (before challenge), HDEP % exhibited the most significant enhancement (*p* < 0.05) in hens administered PomNEs at levels of 1.2 and 0.6 g/kg, with no statistically significant differences between the two treatments, compared with the NC and IC groups. During early and peak egg production phases (before challenge), hens administered PomNEs at a concentration of 1.2 g/kg had the least substantial (*p* < 0.05) FCR and the lowest (*p* < 0.05) NFEI and egg mass percentage, unlike the control groups.

At the conclusion of the mid-laying period (after challenge), the diminished HDEP %, egg mass %, NFEI, and poorer feed conversion ratio (FCR) in groups challenged with *S. Typhimurium* were substantially (*p* < 0.05) ameliorated among groups offered dietary PomNEs at different concentrations. Throughout the mid-laying period (after challenge), hens provided with PomNEs1.2 and PomNEs0.6 exhibited significant (*p* < 0.05) enhancements in HDEP and egg mass % with the lowest FCR, with no marked variations among them, in contrast to the IC group. During the mid-period of egg production (after challenge), NFEI data were considerably elevated (*p* < 0.05) in the PomNEs1.2 group, followed by PomNEs0.6 and PomNEs0.3 groups, unlike the IC group.

**Figure 3:**
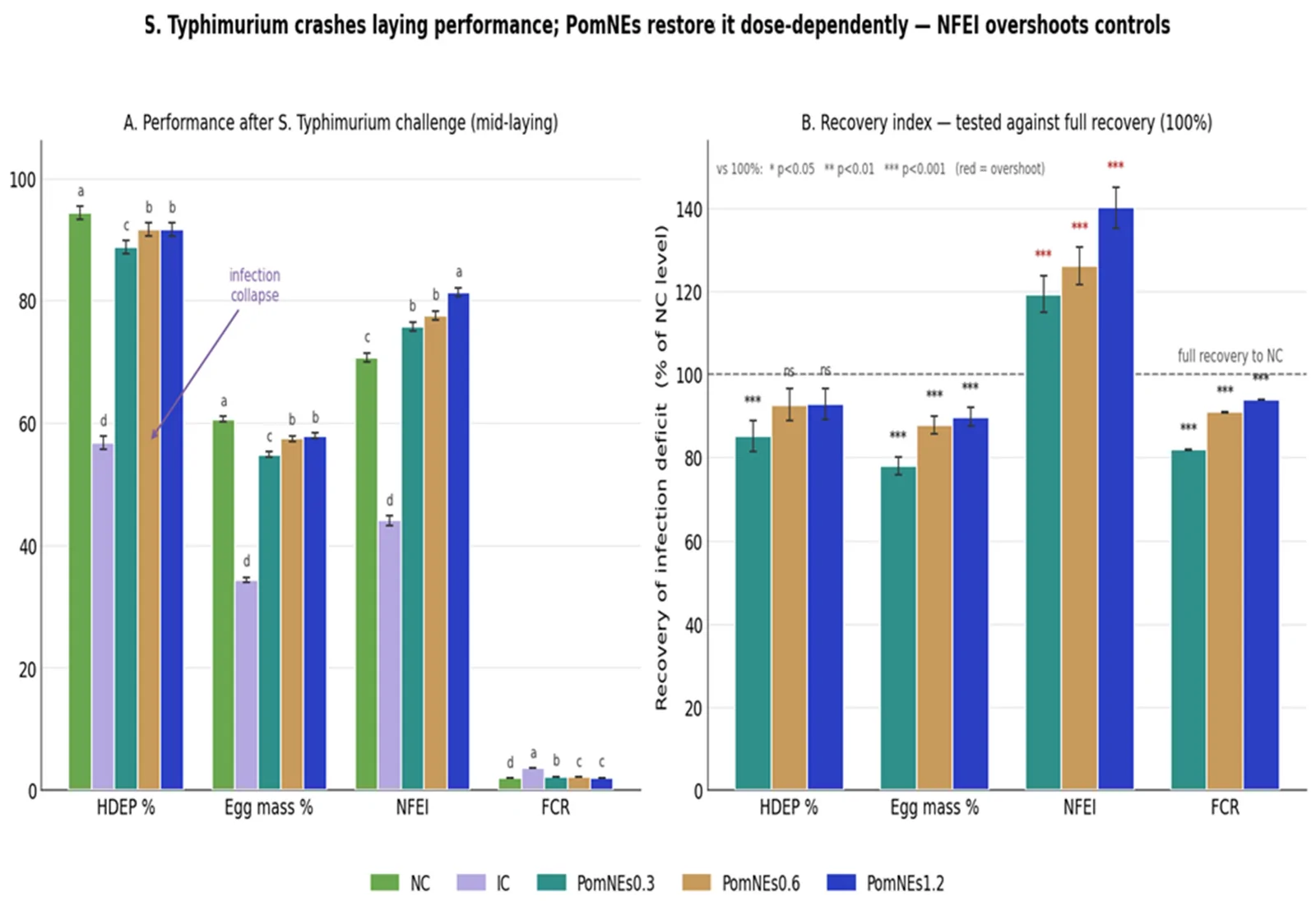
Absolute laying performance and recovery index in mid-laying Hy-Line Brown hens challenged with multidrug-resistant *S. Typhimurium* and fed graded dietary levels of pomegranate peel extract-loaded nano-emulsions (PomNEs). (**A**) Hen-day egg production (HDEP %), egg mass %, net feed efficiency index (NFEI), and feed conversion ratio (FCR) across the five experimental groups after challenge. Data are presented as means ± SEM (bars). Within each performance trait, different superscript letters (a–d) denote significant differences among groups (*p* < 0.05). (**B**) Recovery index for each PomNE dose, plotted for HDEP %, egg mass %, NFEI, and FCR. The recovery index expresses the percentage of the infection-induced deficit (the gap between the infective control [IC] and negative control [NC]) restored toward the NC level; the dashed line marks complete recovery to the NC level (100%). Each value was tested against the 100% reference using a one-sample z-test [z = (R − 100)/propagated SE]: asterisks denote a significant difference from full recovery *** *p* < 0.001), “ns” denotes no significant difference from full recovery, and red asterisks denote values significantly above the NC level. NC (negative control): hens fed a basal diet without additives and not challenged. IC (infective control): hens fed a basal diet without additives and challenged with *S. Typhimurium*. PomNEs0.3, PomNEs0.6, and PomNEs1.2: hens fed a basal diet supplemented with PomNEs at 0.3, 0.6, and 1.2 g/kg diet, respectively.

The relation between the dose of PomNEs added and the recovery in laying performance is illustrated in Figure 3. HDEP recovery reached 93% of the healthy level at the two higher doses, FCR recovery reached 94%, and egg mass recovery reached 90%, whereas NFEI exceeded the uninfected control level, with recoveries of 119%, 126%, and 140% at the three ascending doses. The recovery is itself dose-dependent: PomNEs0.3 restores most of the deficit, but PomNEs0.6 and PomNEs1.2 push performance back to the healthy baseline. Because the recovery index is a ratio of mean differences, its stability decreases when the deficit between the negative and infected controls (the denominator) is small, particularly those near or above 100%, as with the NFEI.

### 3.2. Analysis of Immunological-Related Blood and Serum Biomarkers

As indicated in Table 4, the immunological response subsequent to PomNE fortification was evaluated at the conclusion of the peak laying phase (prior to *S. Typhimurium* challenge, at the end of 33 weeks) and at the end of the mid-laying phase (following *S. Typhimurium* challenge, at the end of 45 weeks). Before the *S. Typhimurium* challenge, our findings revealed that administering PomNEs to hens exerted a substantial immune-stimulating effect (*p* < 0.05) on phagocytic percentage, intracellular killing capability percentage, and lysozyme activity in a dose-dependent manner. Moreover, groups administered PomNEs at a level of 1.2 g/kg had the most substantial (*p* < 0.05) immunological reaction, evidenced by elevated phagocytic percentage, intracellular killing capability percentage, and serum lysozyme activity, unlike the control group. Hens fed PomNEs at concentrations of 1.2 and 0.6 g/kg showed the most significant decrease in serum concentrations of NO, unlike the control group, at the end of 33 weeks.

Following *S. Typhimurium* infection, the phagocytic % rose significantly (*p* < 0.05) in PomNE groups, with no significant variations among the treatments and the NC group compared to the IC group. Following the *S. Typhimurium* challenge, hens receiving PomNEs exhibited a considerable (*p* < 0.05) elevation in intracellular killing capacity and serum lysozyme activity, alongside a marked (*p* < 0.05) reduction in NO level, in a dose-related effect, unlike the IC group. The group of hens administered PomNEs1.2 demonstrated the highest considerable (*p* < 0.05) intracellular killing capacity and serum lysozyme concentrations, alongside the lowest level of inflammatory serum NO compared to the IC group at the end of 45 weeks following the *S. Typhimurium* challenge.

### 3.3. Quantitative Real-Time PCR Test for Salmonella Typhimurium DNA Copy Counting

**Figure 4:**
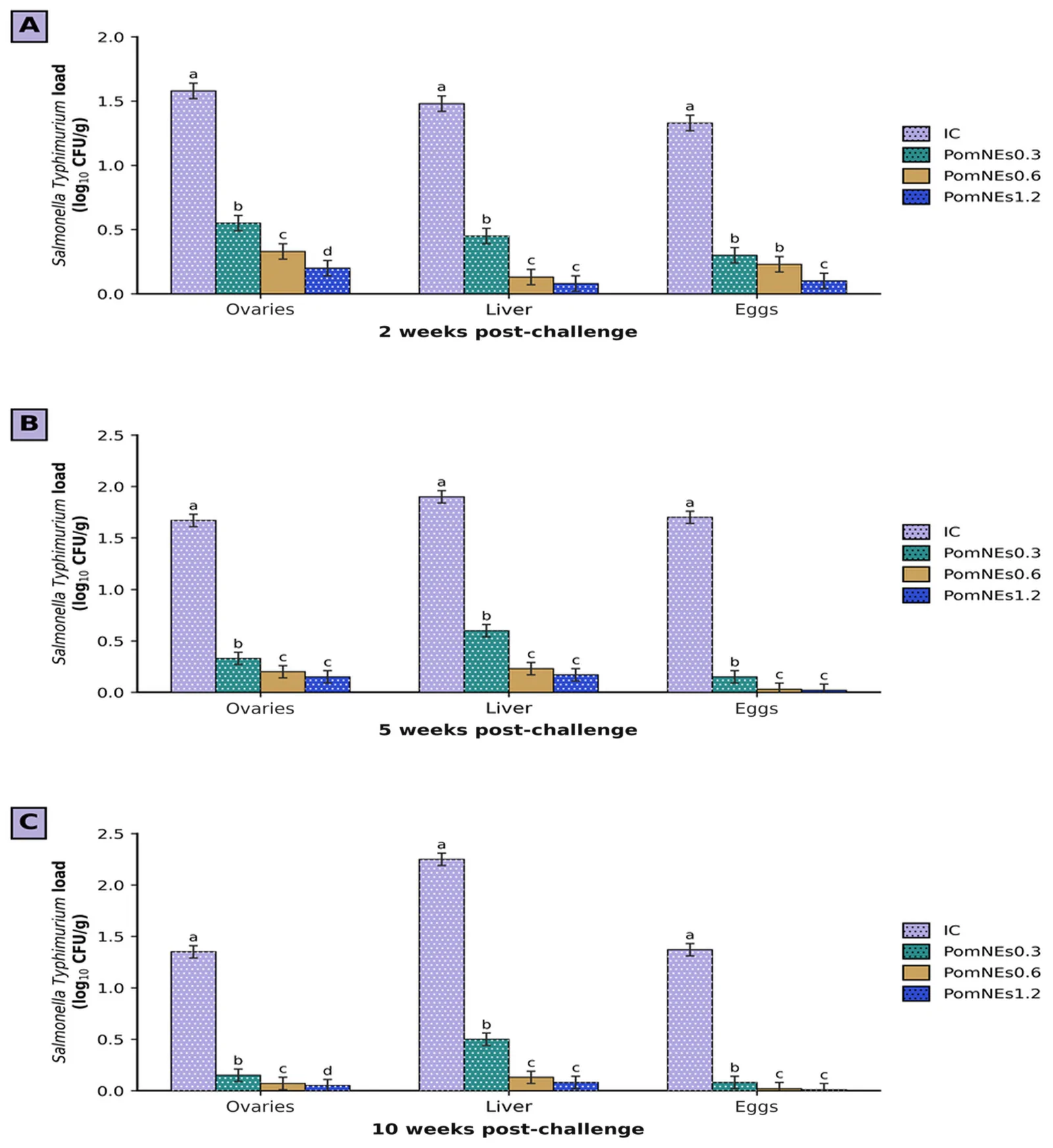
Quantification of *Salmonella Typhimurium* abundance in the ovaries, liver, and eggs of Hy-Line Brown laying hens receiving dietary pomegranate peel extract-loaded nano-emulsions (PomNEs) (**A**) at 2, (**B**) 5, and (**C**) 10 weeks post-challenge with the *S. Typhimurium* strain utilizing the qPCR technique. Results are depicted as means ± SEM (standard error of the mean, in bars). IC (infective control): hens received a basic diet free of additives and were infected with *S. Typhimurium*. PomNEs0.3, PomNEs0.6, PomNEs1.2: hens were provided a basic diet augmented with dietary PomNEs at levels of 0.3, 0.6, and 1.2 g/kg diet, respectively. At 34 weeks of age (mid-laying period), the IC and three treatment groups were infected with the MDR *S. Typhimurium* strain. ^a–d^ Variable superscript letters imply significant differences (*p* < 0.05) in means within the same column.

The quantification findings of *S. Typhimurium* in the ovaries, liver, and eggs of Hy-Line Brown laying hens at 2, 5, and 10 weeks post-challenge with the MDR *S. Typhimurium* strain are depicted in Figure 4. Significant *S. Typhimurium* colonization in the eggs, ovaries, and liver was observed in the IC group at 2, 5, and 10 weeks post-challenge. The quantitative analysis of *S. Typhimurium* in the ovaries, liver, and eggs of challenged hens demonstrated that all PomNE-treated groups had considerably (*p* < 0.05) reduced Salmonella loads relative to the IC group, with a consistent decrease in loads over time. The inclusion of dietary PomNEs markedly (*p* < 0.05) minimized *S. Typhimurium* populations in the ovaries, liver, and eggs at 2, 5, and 10 wpi compared to the IC group. At 2 and 10 wpi, *S. Typhimurium* colonization in the ovaries was considerably reduced (*p* < 0.05) in a dose-dependent way following PomNE administration compared to the infected and supplemented control group. At 5 wpi, the *S. Typhimurium* abundance was markedly reduced in the ovaries, liver, and eggs of challenged hens receiving PomNEs1.2 and PomNEs0.6, with no significant variations among them, in comparison to the IC group. At 2 wpi, hens offered PomNEs1.2 showed the most substantial (*p* < 0.001) decrease in *S. Typhimurium* load in their eggs compared to the IC group. Furthermore, at 2 and 10 wpi, hens receiving a diet fortified with PomNEs1.2 (0.09 log10 CFU/g each) and PomNEs0.6 exhibited the most rapid recovery from *S. Typhimurium* colonization in the liver, with no significant variations among the two treatments, compared with the IC group. Interestingly, no *S. Typhimurium* CFUs were identified in the eggs and ovaries of hens administered PomNEs at a dosage of 1.2 g/kg at 10 weeks post-challenge with *S. Typhimurium*. Moreover, a remarkable reduction in Salmonella populations in eggs was observed in challenged hens offered PomNEs0.6 and PomNEs1.2 at 10 wpi, displaying their protective roles against *Salmonella* infection.

### 3.4. Quantitative Analysis of Virulence Gene Expression in Salmonella Typhimurium

**Figure 5:**
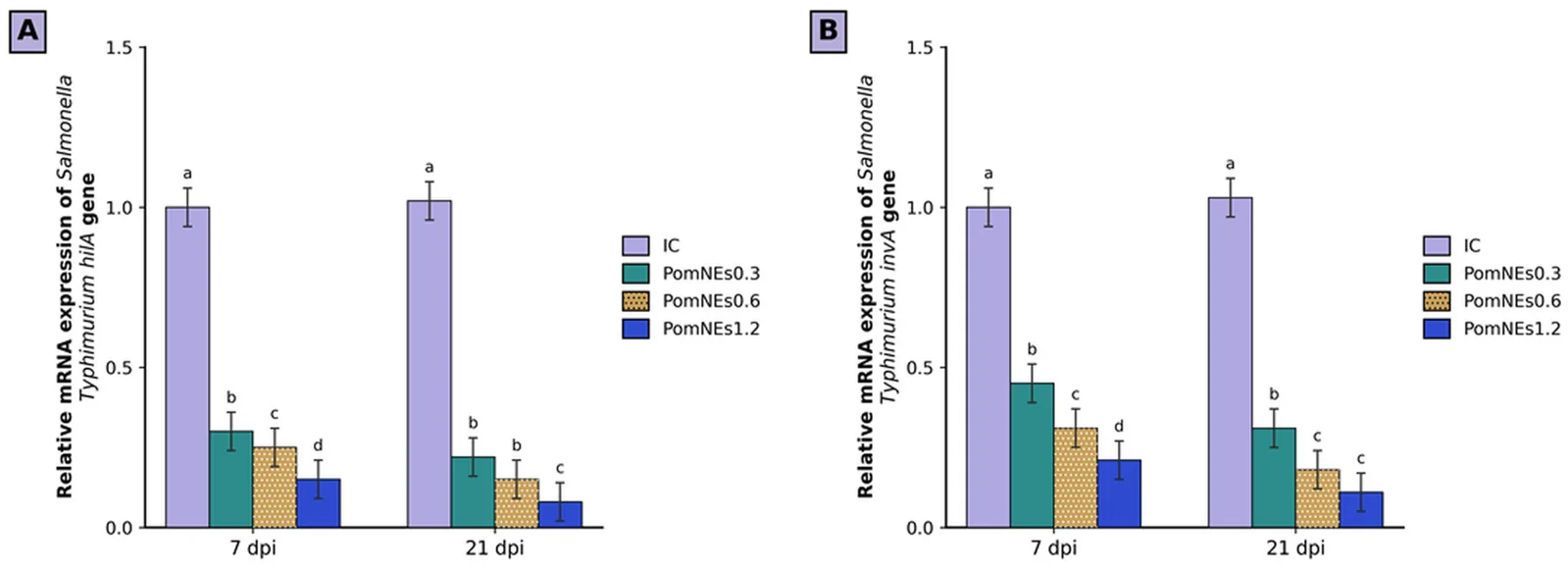
mRNA transcript levels of virulence-related genes in *Salmonella Typhimurium*, invasion protein regulator (*hilA*, (**A**)), and invasion protein A (*invA*, (**B**)), in the intestinal tissues of challenged Hy-Line Brown laying hens offered dietary pomegranate peel extract-loaded nano-emulsions (PomNEs) at 7 and 21 days post-infection (dpi) with *S. Typhimurium*, as estimated by RT-qPCR. Results are depicted as means ± SEM (standard error of the mean, in bars). IC (infective control): hens received a basic diet free of additives and were infected with *S. Typhimurium*. PomNEs0.3, PomNEs0.6, PomNEs1.2: hens were provided a basic diet augmented with dietary PomNEs at levels of 0.3, 0.6, and 1.2 g/kg diet, respectively. At 34 weeks of age (mid-laying period), the IC and three treatment groups were infected with the MDR *S. Typhimurium* strain. ^a–d^ Variable superscript letters imply significant differences (*p* < 0.05) in means within the same column.

Figure 5 demonstrates the impact of dietary PomNEs on the expression of *S. Typhimurium invA* and *hilA* virulence genes in the intestinal tissues of challenged hens, evaluated by RT-qPCR at 7 and 21 days post-infection with the MDR *S. Typhimurium* strain. The findings demonstrated that PomNEs substantially (*p* < 0.05) decreased the expression levels of *S. Typhimurium invA* and *hilA* invasion genes, in a dose-dependent way, in contrast to the IC group at 7 dpi with S. Typhimurium. The dietary incorporation of PomNEs1.2 and PomNEs0.6 led to substantial (*p* < 0.05) down-regulation of the *S. Typhimurium* virulence *invA* gene, with no substantial variations between them, at 21 dpi. In comparison to the IC group, the most significant (*p* < 0.05) reduction in *S. Typhimurium* *hilA* transcription levels was detected in the intestinal tissues of birds fed with PomNEs1.2, followed by PomNEs0.6 and PomNEs0.3 at 21 dpi.

### 3.5. Relation Between PomNE Dose and Pathogen Clearance and Virulence-Gene Silencing

**Figure 6:**
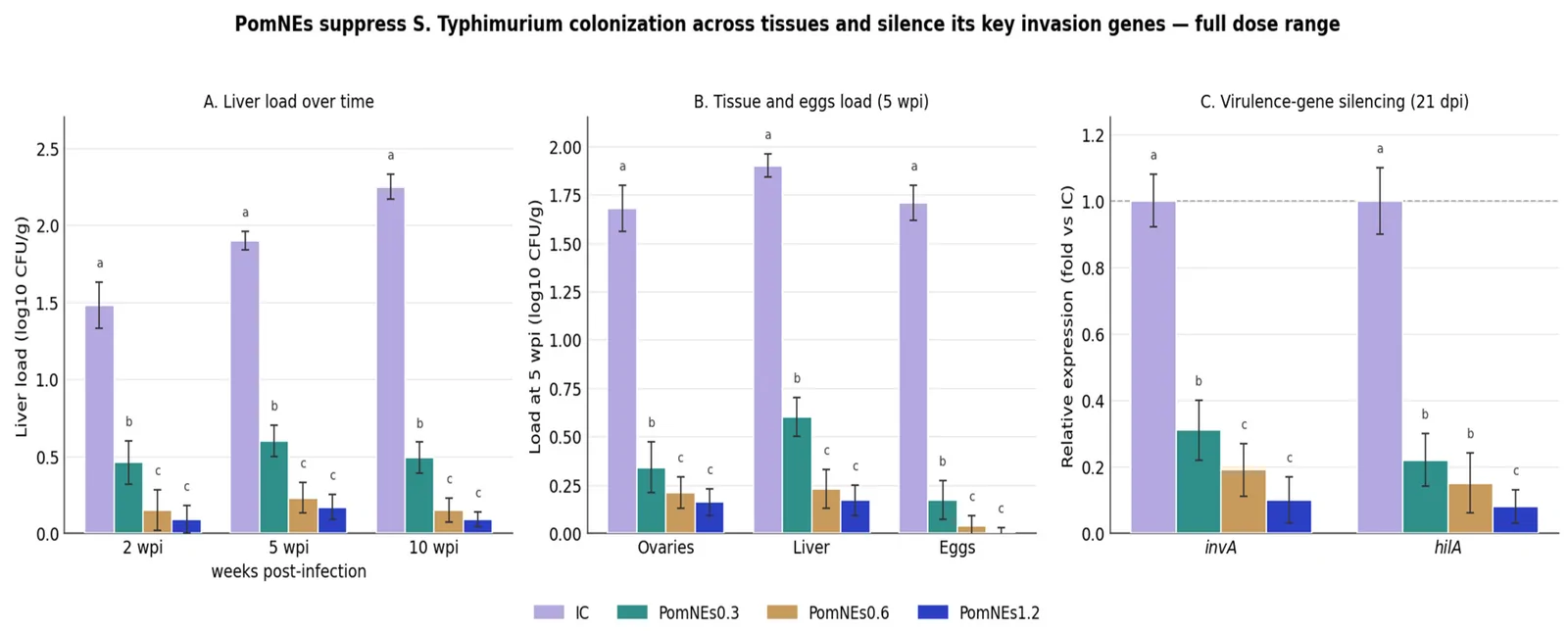
Pathogen clearance kinetics, tissue burden, and virulence-gene silencing across the full PomNE dose range (IC, PomNEs0.3, PomNEs0.6, PomNEs1.2). (**A**) Liver clearance kinetics. (**B**) Ovaries, Liver, and eggs loads at 5 weeks post-infection. (**C**) *invA* and *hilA* expression at 21 days post-infection. IC (infective control): hens received a basic diet free of additives and were infected with *S. Typhimurium*. PomNEs0.3, PomNEs0.6, PomNEs1.2: hens were provided a basic diet augmented with dietary PomNEs at levels of 0.3, 0.6, and 1.2 g/kg diet, respectively. ^a–c^ Variable superscript letters imply significant differences (*p* < 0.05) in means within the same column.

Figure 6 addresses the microbiological side of PomNEs; they reduce the pathogen itself and deactivate its machinery. Regarding clearance kinetics (Figure 6A), in the IC group, liver colonization worsens over time (1.48 → 2.25 log10 CFU/g from 2 to 10 weeks). In treated birds, the load stays low and flat. Tissue and egg burden (Figure 6B): at 5 weeks, PomNEs reduce loads by roughly tenfold across the ovaries, liver, and eggs. In eggs, PomNEs1.2 drives the count near to zero. Anti-virulence (Figure 6C): *invA* and *hilA* are silenced to 0.08–0.10-fold relative to the IC at the highest dose, again dose-dependently. Together, these panels show two complementary actions: a quantitative reduction in *S. Typhimurium* and a qualitative reduction in its virulence genes.

### 3.6. Quantitative Analysis of Immune-Related Gene Expression in Response to Salmonella Typhimurium Challenge

**Figure 7:**
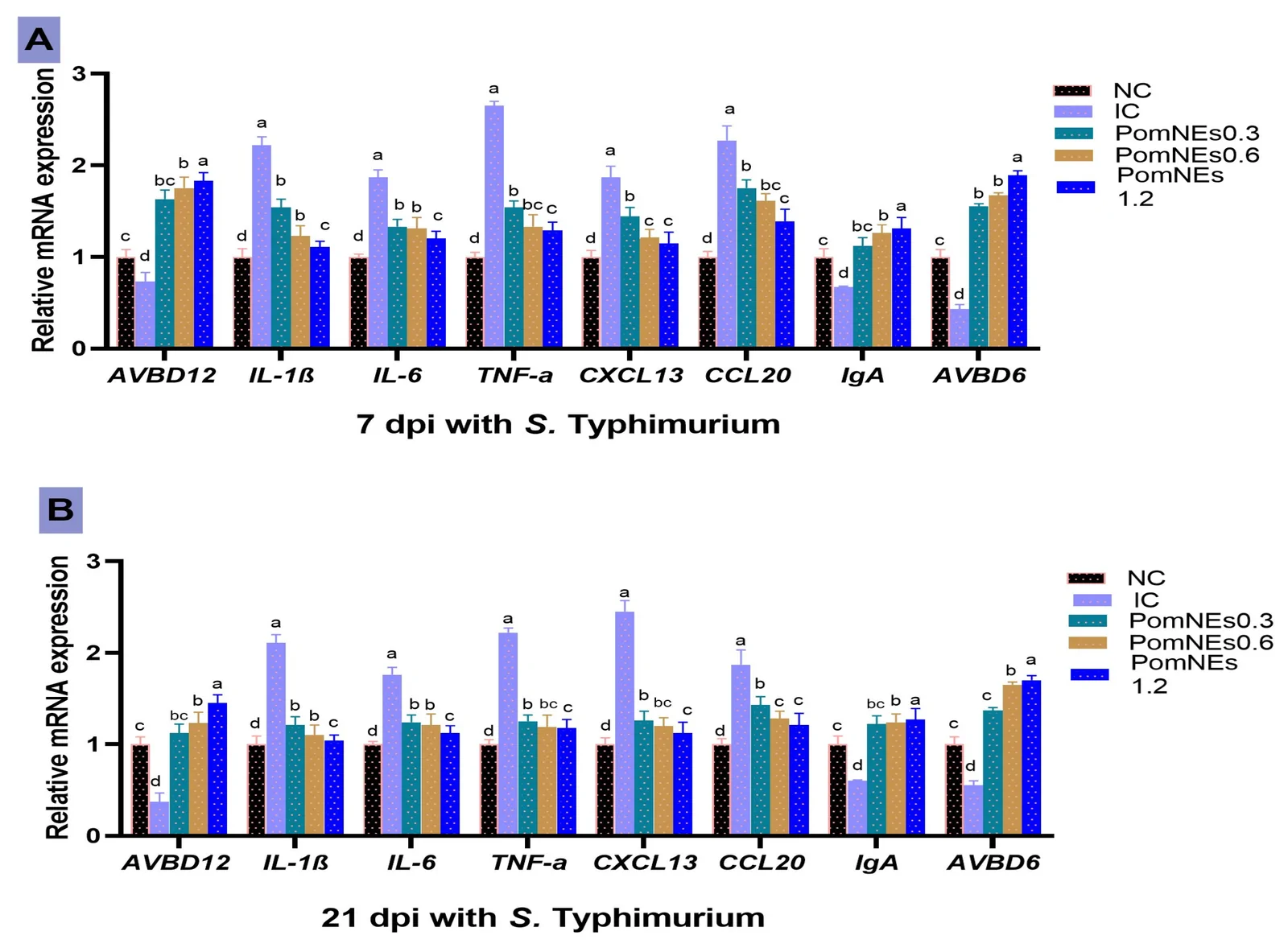
The efficacy of dietary inclusion with pomegranate peel extract-loaded nano-emulsions (PomNEs) on the mRNA transcript levels of immune response-related genes [interleukins (*IL-1β, IL-6*), tumor necrosis factor (*TNF-α*), C–C motif ligand 20 (*CCL20*), stromal cell-derived factor-1 (*CXCL13*), immunoglobulin A (*IgA*), and Avian β-defensin 6 and 12 (*AvBD6* and *AvBD12*)] in the intestinal tissues of Hy-Line Brown laying hens at 7 (**A**) and 21 (**B**) days post-infection (dpi) with *S. Typhimurium* (mid-laying period) via RT-qPCR assay. Results are depicted as means ± SEM (standard error of the mean, in bars). NC (negative control): hens received a basic feed lacking any supplements and were not exposed to any challenges. IC (infective control): hens received a basic diet free of additives and were infected with *S. Typhimurium*. PomNEs0.3, PomNEs0.6, PomNEs1.2: hens were provided a basic diet augmented with dietary PomNEs at levels of 0.3, 0.6, and 1.2 g/kg diet, respectively. At 34 weeks of age (mid-laying period), the IC and three treatment groups were infected with the MDR *S. Typhimurium* strain. ^a–d^ Variable superscript letters imply significant differences (*p* < 0.05) in means within the same column.

Varying expression levels of genes related to intestinal immune response at 7 and 21 days post-infection with *S. Typhimurium* (mid-laying period) as influenced by the inclusion of dietary PomNEs are shown in Figure 7. At 7 and 21 dpi with *S. Typhimurium*, dietary inclusion with PomNEs markedly (*p* < 0.05) minimized the elevated inflammatory response and augmented the reduced intestinal protective responses following *S. Typhimurium* challenge. At 7 and 21 days post-infection with *S. Typhimurium*, the inclusion of dietary PomNEs markedly (*p* < 0.05) reduced the transcript levels of genes related to pro-inflammatory chemokines (*CCL20* and *CXCL13*) and cytokines (*IL-1β, TNF-α*, and *IL-6),* besides increasing transcript levels of intestinal protective genes, including antimicrobial peptides (*AvBD6* and *AvBD12*) and *IgA*, in comparison to the infected control group. At 7 and 21 dpi, the most pronounced up-regulation (*p* < 0.05) of the *IgA* gene along with the intestinal innate antimicrobial defense genes, specifically *AvBD6* and *AvBD12*, was observed in the group supplemented with PomNEs1.2 compared to the infected control group. At both time points, the transcription levels of *IL-1β* and *IL-6* genes were reduced by increasing doses of PomNEs, attaining their lowest levels in the PomNEs1.2 group. The expression of *TNF-α*, *CCL20*, and *CXCL13* genes was significantly down-regulated (*p* < 0.05) in hens offered dietary PomNEs1.2, followed by those administered PomNEs0.6, with no notable variations among the two treatment groups at both time points.

### 3.7. Regulation of Antioxidant-Related Genes in Response to Dietary Pomegranate Peel Extract-Loaded Nano-Emulsions and S. Typhimurium Challenge

**Figure 8:**
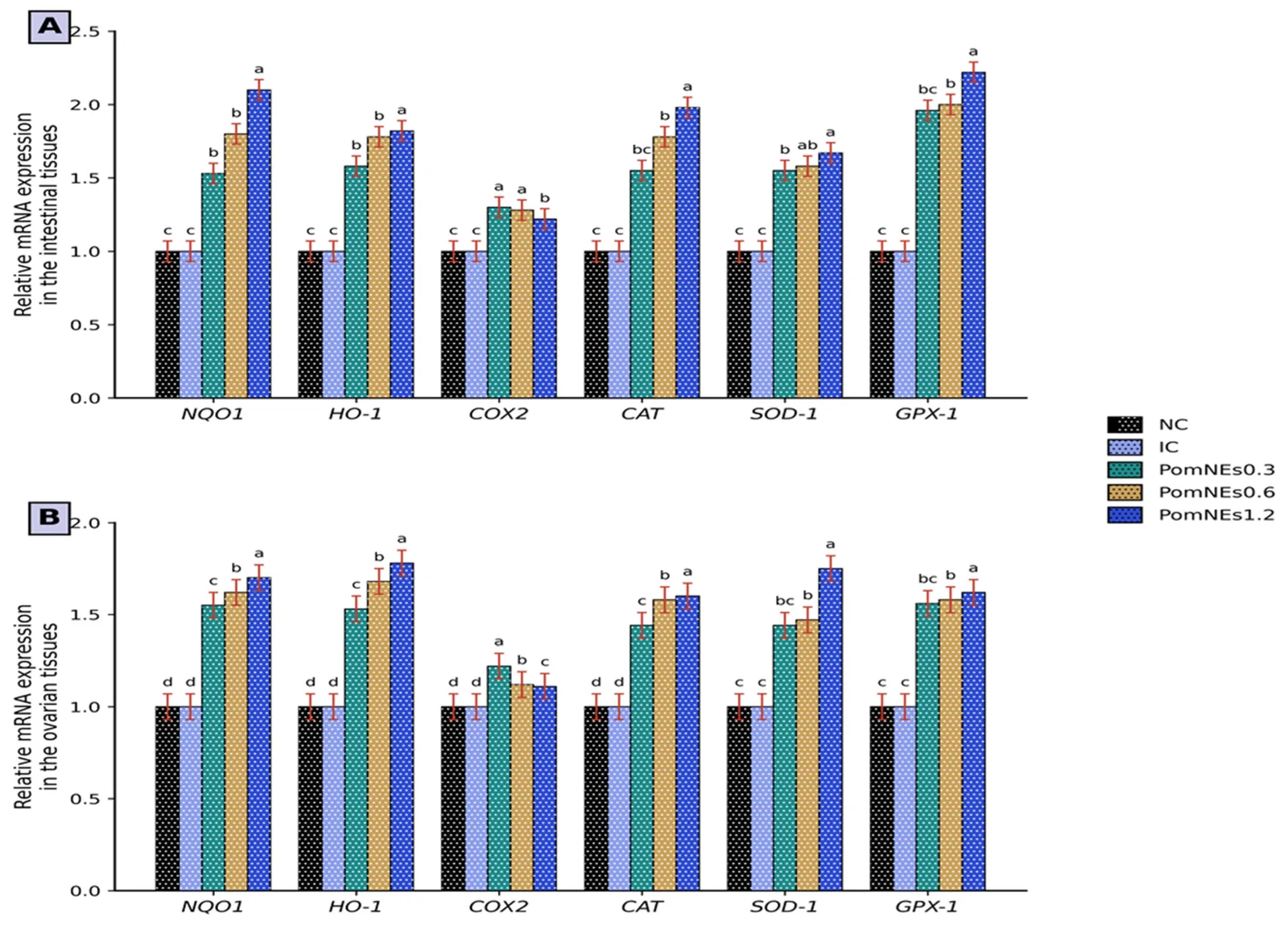
Impact of dietary inclusion of pomegranate peel extract-loaded nano-emulsions (PomNEs) on the mRNA expression of antioxidant-encoding genes—haemoxygenase-1 (*HO-1*), cyclooxygenase-2 *(COX-2*), NAD(P)H dehydrogenase quinone 1 (*NQO1*), catalase (CAT), superoxide dismutase (*SOD-1*), and superoxide glutathione peroxidase (*GPX-1*)—in the intestinal (**A**) and ovarian (**B**) tissues of Hy-Line Brown laying hens at 33 weeks of age (end of peak laying period, before *S. Typhimurium* challenge) via RT-qPCR. Results are depicted as means ± SEM (standard error of the mean, in bars). NC (negative control): hens received a basic feed lacking any supplements and were not exposed to any challenges. IC (infected control): hens received a basic diet free of additives and were infected with *S. Typhimurium*. PomNEs0.3, PomNEs0.6, PomNEs1.2: hens were provided a basic diet augmented with dietary PomNEs at levels of 0.3, 0.6, and 1.2 g/kg diet, respectively. At 34 weeks of age (mid-laying period), the IC and three treatment groups were infected with the MDR *S. Typhimurium* strain. ^a–d^ Variable superscript letters imply significant differences (*p* < 0.05) in means within the same column.

Data concerning the effect of dietary supplementation with graded doses of PomNEs on the transcription of antioxidant-encoding genes in the intestinal and ovarian tissues of hens at 33 weeks of age (before *S. Typhimurium* challenge at the conclusion of the peak laying phase) are emphasized in Figure 8. The expression of intestinal and ovarian *CAT*, *HO-1*, *GPX-1*, *NQO1*, and *SOD-1* genes was considerably raised (*p* < 0.05) in the PomNEs groups, along with a significant (*p* < 0.05) reduction in the expression of intestinal and ovarian *COX2*, compared to the control group. The mRNA transcription of intestinal *SOD-1* was considerably elevated (*p* < 0.05) in the groups administered PomNEs1.2 and PomNEs0.6, with no substantial variations among the two treatment groups, in contrast to the control group. Additionally, the highest expressions (*p* < 0.05) of intestinal and ovarian *CAT*, *GPX-1*, *HO-1*, *NQO1*, and ovarian *SOD-1* genes were documented in the PomNEs1.2 augmented group. PomNE inclusion notably (*p* < 0.05) boosted the transcript levels of ovarian CAT, *NQO1*, and *HO-1* in a dose-dependent way. Simultaneously, the lowest expression level of intestinal and ovarian *COX2* was significantly (*p* < 0.05) seen in the PomNEs1.2 group.

**Figure 9:**
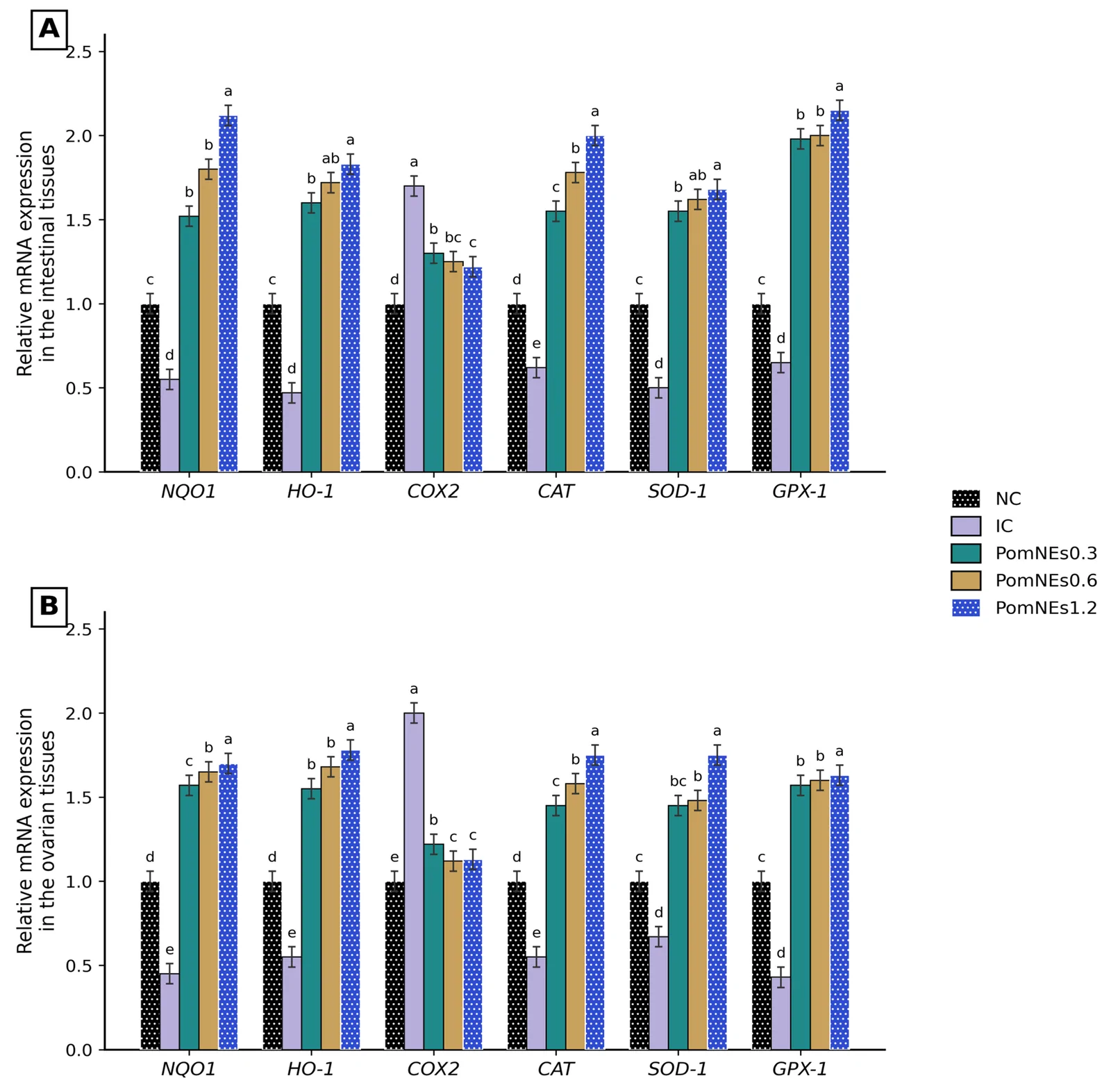
RT-qPCR analysis of the relative expression levels of antioxidant-related genes—haemoxygenase-1 (*HO-1*), *NAD(P)H* dehydrogenase quinone 1 (*NQO1*), cyclooxygenase-2 (*COX-2*), catalase (*CAT*), superoxide dismutase (*SOD-1*), and superoxide glutathione peroxidase (*GPX-1*)—in the intestinal (**A**) and ovarian (**B**) tissues of challenged Hy-Line Brown laying hens supplemented with dietary pomegranate peel extract-loaded nano-emulsions (PomNEs) at 45 weeks of age (end of mid-laying period) following *S. Typhimurium* challenge. Results are depicted as means ± SEM (standard error of the mean, in bars). NC (negative control): hens received a basic feed lacking any supplements and were not exposed to any challenges. IC (infected control): hens received a basic diet free of additives and were infected with *S. Typhimurium*. PomNEs0.3, PomNEs0.6, PomNEs1.2: hens were provided a basic diet augmented with dietary PomNEs at levels of 0.3, 0.6, and 1.2 g/kg diet, respectively. At 34 weeks of age (mid-laying period), the IC and three treatment groups were infected with the MDR *S. Typhimurium* strain. ^a–d^ Variable superscript letters imply significant differences (*p* < 0.05) in means within the same column.

Figure 9 illustrates the transcription levels of antioxidant-encoding genes in the intestinal and ovarian tissues of challenged hens supplemented with varying concentrations of PomNEs at 45 weeks of age (conclusion of mid-laying phase) following *S. Typhimurium* infection. Dietary supplementation with PomNEs alleviated the adverse effects of *S. Typhimurium* challenge on antioxidant-encoding genes, restoring their functions to degrees similar to those in the NC group. The dietary PomNE supplementation considerably (*p* < 0.001) enhanced the expression levels of intestinal and ovarian *CAT*, *NQO1*, *SOD-1*, *HO-1*, and *GPX-1*, along with reducing the transcript level of intestinal and ovarian *COX2* following *S. Typhimurium* challenge, unlike the infected control group. Notably, the administration of dietary PomNEs at a concentration of 1.2 g/kg in challenged hens resulted in the most substantial (*p* < 0.001) up-regulation in intestinal and ovarian *NQO1*, *CAT*, and *GPX-1*, as well as ovarian *HO-1* and *SOD-1* genes, and the most notable down-regulation in the ovarian *COX2* gene expression level compared to the IC group. Furthermore, administering PomNEs to challenged hens at dosages of 1.2 and 0.6 g/kg significantly (*p* < 0.001) elevated the transcription levels of intestinal ovarian *HO-1* and *SOD-1* genes, and reduced the intestinal *COX2* gene transcription level, with no considerable variances among the two treatments, compared to the IC group.

## 4. Discussion

Infection with *S. Typhimurium*, especially the MDR strain, has caused considerable harm to the poultry industry because of decreased egg production, poor feed efficiency in laying hens, and increased mortality and stunted growth in broilers, as well as its link to human food-borne diseases [13]. Thus, there is a pressing need to implement efficient measures to manage salmonellosis in poultry production, including the use of phytogenics [43]. Prior research effectively employed phytogenics to reduce *S. Typhimurium* in challenged laying hens and promote their productivity [44]. Nonetheless, the impact of dietary phytogenics-loaded nano-emulsions, including PomNEs, on reducing *S. Typhimurium* infection in challenged hens remains unexplored. Given this gap, the present research aims to study, for the first time, the efficacy of dietary PomNEs on laying performance, immunity, antioxidant competence, and *S. Typhimurium* resistance in challenged hens. In this investigation, hens offered dietary PomNEs1.2 demonstrated the maximum hen-day egg production percentage (HDEP%) and net feed efficiency index (NFEI), as well as an improved feed conversion ratio (FCR) during the early and peak laying phases (before challenge) compared to the control groups. Dietary polyphenols such as those in pomegranate peel are chemically fragile as they are degraded by low gastric pH, digestive enzymes, oxygen, and light—also, their poor water solubility and astringency limit how much reaches the intestine in an absorbable form [45]. Nano-encapsulation overcomes this by enclosing the polyphenols behind an oil phase and an interfacial film that shield them from acid and enzymatic attack and delay release until the intestinal phase [46]. The superior egg production observed in the current study could be attributed to the advantages of the nanoscale form of pomegranate peel extract. Its large surface area enhances lipid digestion and mixed micelle formation, thereby improving solubilization, mucus penetration, and epithelial uptake. These effects increase the bio-accessibility and oral bioavailability of the compounds, ultimately contributing to improved laying performance [47]. A two-stage (double, W_1_/O/W_2_) system extends this protection by sequestering the hydrophilic polyphenols behind two successive interfaces rather than one, which measurably increases encapsulation efficiency and preserves phenolic content and antioxidant activity through simulated digestion [45,46]. Compared with a single nano-emulsion, it is simpler, smaller, and more stable, but leaves active sites near the interface exposed and prone to burst. The dual design trades some droplet size and stability for stronger acid/enzyme protection, higher loading, more sustained intestinal release, and better astringency masking [25]. This is precisely the architecture used for the present pomegranate peel extract, which plausibly explains its sustained in vivo antioxidant, immunomodulatory, and anti-*Salmonella* effects, consistent with comparable pomegranate and phytogenic nano-emulsion results in challenged animals [48].

The dietary inclusion of PomNEs, particularly at higher levels, markedly enhanced HDEP% and NFEI and decreased FCR throughout the mid-laying phase (after challenge) in *S. Typhimurium*-challenged hens compared to the IC group. In agreement, prior studies indicated that dietary essential-oil fortification markedly enhanced egg-laying performance and egg production traits in hens challenged with *S. Enteritidis* [2] and *S. Typhimurium* [44]. Accordingly, prior research demonstrated that dietary phytogenics enhanced FCR and elevated egg production % in *S. pullorum*-challenged laying hens compared to the positive control group [49]. Likewise, the dietary incorporation of pomegranate peel extract markedly improved egg-laying performance in laying hens [19,50]. Nevertheless, this work provides novel insights into the impact of PomNEs on the egg-laying performance of *S. Typhimurium*-infected hens. The augmented egg-laying performance upon dietary phytogenic fortification may account for the ability of their flavonoid polyphenols to improve the hens’ immune response, appetite, food consumption, and digestibility, while also exhibiting antioxidant and antimicrobial properties [17]. The enhanced effectiveness of PPE on egg-laying performance results from its application as a polymeric nano-emulsion, which regulates the dispersal of its bioactive components and enhances its bioavailability and stability [51]. The gains in laying performance, including higher hen-day egg production, a superior net feed efficiency index and feed conversion ratio, and maintained egg mass, were dose-dependent (maximal at 1.2 g/kg) and, importantly, appeared before the *S. Typhimurium* challenge, indicating a primary nutritional and physiological benefit rather than a consequence of infection mitigation alone. Mechanistically, the double nano-emulsion likely protects the principal peel polyphenols (punicalagin and ellagic acid) from premature degradation in the upper gastrointestinal tract and increases their intestinal residence and cellular uptake, delivering a more bioavailable and sustained polyphenol dose to the enterocyte, which reasonably accounts for the graded, dose-responsive effect [52]. At the mucosal level, these polyphenols reinforce epithelial architecture and improve nutrient digestibility, an effect that translates directly into the observed feed-efficiency and egg-production improvements [29]. Concurrently, their antioxidant activity lowers the metabolic cost of counteracting epithelial oxidative turnover, sparing metabolizable energy and amino acids that can be re-partitioned toward yolk-precursor synthesis and sustained ovulation, consistent with the elevated hen-day egg production and egg mass. Following challenge, these same barrier-protective and antioxidant actions shield the reproductive tract from *Salmonella*-driven disruption, explaining why performance was preserved in supplemented hens rather than deteriorating as in the infected control; the greater-than-complete net feed efficiency recovery is congruent with this pre-existing performance-promoting effect superimposed on infection protection.

Avian salmonellosis, particularly invasive *S. Typhimurium* infections, is usually started by oral intake of the bacterium, which causes gut colonization and quickly spreads to internal organs, including the ovaries and liver. As a result, eggs may become contaminated with bacteria. *S. Typhimurium* contamination presents a considerable risk to the egg-laying performance and well-being of hens, highlighting the necessity of efficient alternative preventative measures, such as phytogenics [53]. *S. Typhimurium* quantitative analytical results in the ovaries, liver, and eggs of hens post-challenge indicated that the incorporation of dietary PomNEs consistently diminished *S. Typhimurium* abundance in the ovaries, liver, and eggs of challenged hens at 2, 5, and 10 weeks post-challenge with the MDR *S. Typhimurium* strain, unlike the infective control group, suggesting their antimicrobial activities. Previous studies demonstrated in vitro antibacterial activities of PomNEs against *S. Typhimurium* [22]. In accordance, dietary phytogenic inclusion markedly minimized *S. Typhimurium* populations in the crop, gizzard, duodenum, and ceca of hens challenged with *S. Typhimurium* [44]. Furthermore, supplementation with pomegranate peel extract at higher doses (3 and 6 g/kg) was able to reduce lesion scores and oocyst shedding and restore growth in Eimeria-infected broilers, with 6 g/kg identified as the most effective dose [54]. Likewise, incorporation with dietary phytogenics, either in free [55,56] or loaded nano-emulsion [57] forms, reduced *S. Typhimurium* loads in challenged broilers; nevertheless, the effect of dietary PomNE inclusion on *S. Typhimurium* abundance in the ovaries, liver, and eggs of challenged hens remains uninvestigated. Parallel gains have been documented in complex food matrices, where nano-encapsulated PPE preserved phenolic content and delivered superior antioxidant and antimicrobial protection compared with the free extract, owing to the enhanced stability and functionality of the delivered polyphenols [58].

These outcomes may be accounted for by cell wall fragmentation, injury to membrane proteins and the cytoplasmic membrane, a decrease in the cytoplasmic coagulation, proton motive force, and leakage of cellular components [43]. Concerning *S. Typhimurium* resistance in challenged hens following the incorporation of dietary PomNEs, previous research has demonstrated that phytogenics can alter innate immunity by reducing bacterial viability, ameliorating NO synthesis, and boosting the phagocytic capability of macrophages [59].

Nutritional immunology represents a novel strategy in managing microbial diseases in the poultry industry, circumventing the constraints of vaccination tactics by employing feed additives [16]. Furthermore, improving the diet and medical care of poultry will make their rearing more cost-effective and efficient by minimizing illnesses; hence, many treatment options presently focus on the pathogenicity of bacteria rather than the survival of bacteria [60]. *Salmonella Typhimurium* pathogenicity is manifested by three principal mechanisms: colonization, invasion, and intracellular survival, with its severity contingent upon several virulence-related variables. It utilizes these virulence attributes to infiltrate the gut epithelial cells and endure within mucosal macrophages, frequently resulting in the onset of an acute inflammatory reaction [61,62]. Natural anti-virulence compounds, such as phytogenics, can regulate virulence indicators that influence disease. Phytogenics have been demonstrated to modify the expression of virulence genes, which are recognized as critical components in the *Salmonella* infection course [63]. Consequently, the anti-virulence properties of PomNEs were evaluated by quantifying the expression values of the *S. Typhimurium invA* and *hilA* virulence-associated genes after their administration at 7 and 21 days post-infection with the MDR *S. Typhimurium* strain. Herein, the most significant decrease in the transcription of *S. Typhimurium invA* and *hilA* genes occurred in the intestinal tissues of hens supplemented with PomNEs1.2, followed by PomNEs0.6 and PomNEs0.3, indicating a possible anti-virulence impact. In accordance, previous studies documented the in vitro anti-virulence effects of PPE [64,65] on *S. Typhimurium* virulence genes. Previous studies have documented the in vivo anti-virulence efficacy of phytogenics against *S. Typhimurium* [43,66] and *S. Enteritidis* [1] in challenged broilers; nevertheless, the in vivo anti-virulence efficacy of PomNEs in *S. Typhimurium*-challenged hens remains unexamined. The down-regulation of *hilA* and *invA* was measured at the transcript level and is considered a putative mechanistic correlate rather than direct proof of attenuated virulence. The reduced expression observed here is best explained by the concurrent reduction in tissue and egg colonization of *S. Typhimurium*.

Blood and serum immunological metrics are essential markers that offer substantial insights into the general well-being of poultry, as their defense mechanism primarily regulates their overall health. Bacterial infections trigger systemic inflammation, significantly impairing immunological function, compromising health, and resulting in reduced bird performance [67,68]. Since phagocytic cells are the primary producers of LYZ and NO, elevated levels of these two molecules may be important indicators of inflammation and a reaction to bacterial infection [69]. However, following Salmonella infection, lysozyme levels can behave complexly in the gut, as Salmonella infection often suppresses LYZ production in the intestinal epithelial cells, potentially impairing host defenses [70]. Birds’ first line of defense against diseases is phagocytosis, an innate immunological reaction that involves engulfing and eliminating foreign particles, such as dead cells and pathogens [71]. Key effector tasks carried out by macrophages include phagocytosis, processing and presentation of antigens, and the generation of cytokines [72]. We postulated that the elevated phagocytic % indicates that PomNEs, especially at higher doses, effectively stimulate macrophages due to the ongoing relationship between macrophages and Salmonella. This likely facilitates the diminution of Salmonella subsequent to their dietary integration, aligning with the findings of García-López, Pinos-Rodríguez [44]. Simultaneously, our investigation revealed that the application of dietary PomNEs, particularly PomNEs1.2, improved the immunological reactions of birds against *S. Typhimurium* infection. This was confirmed by the activation of immune-mediated reactions, characterized by reduced serum NO concentration along with a heightened serum LYZ level, together with an augmented phagocytic percentage and intracellular killing capacity both prior to and following *S. Typhimurium* infection. Likewise, a prior work demonstrated that dietary pomegranate peel extract fortification significantly raised serum the LYZ level [73] and decreased serum NO concentrations [74] in broilers relative to the control group. Accordingly, a recent investigation exhibited that dietary PPE significantly enhanced the immune response of rabbits, as evidenced by an increased phagocytic percentage and decreased serum NO level [75]. Dietary phytogenics decreased the elevated serum NO concentration in broilers challenged with *S. Typhimurium* compared to the infective control group [56]; nevertheless, research on the immune-boosting and anti-inflammatory impacts of dietary PomNEs in *S. Typhimurium*-challenged hens is still lacking.

The primary barrier protecting the host from Salmonella infection is the gut mucosa’s immune defense. *Salmonella Typhimurium* utilizes inflammation to enhance its gut colonization in reaction to flagella and lipopolysaccharides [76]. Cytokines are critical regulators of gut inflammation and significantly contribute to the body’s defense against microbial illnesses. Intestinal immune cells are activated to generate cytokines during microbial invasion of the intestinal epithelial cells [16]. Pro-inflammatory cytokines, such as *IL-1β*, *TNF-α*, and *IL-6*, are essential to acute-phase inflammatory processes associated with systemic and metabolic changes [77], while also influencing the reaction of the immune system during microbial infections [78]. Intestinal infections may elevate the expression of *TNF-α*, *IL-1β*, and *IL-6*, hence increasing the permeability of gut epithelium [79]. Chemokines, or chemotactic cytokines, are considered inflammatory proteins synthesized by macrophages, including *CCL4*, *CCL20*, and *CXCL13*, and are essential for regulating the body’s immune response to disease. Neutrophils’ release of *CCL20* promotes inflammation by attracting extra leukocytes to the inflammation site, which in turn triggers macrophage-dependent clearance of inflammation and results in chronic inflammatory illnesses [80]. *CXCL13*, also known as stromal cell-derived factor-1, has a robust chemotactic influence on lymphocytes implicated in inflammation in response to infections [81]. Additionally, the *COX-2* enzyme regulates the physiological conversion of arachidonic acid into inflammatory prostaglandins, which then trigger the production of cytokines [82] and act as pro-inflammatory mediators, which modulate the inflammatory response triggered by invading bacteria. The immune response of the bird significantly depends on peptide defensin, which offers prompt defense against pathogenic bacteria. *AVBD6* and *AVBD12* exhibit lipopolysaccharide-neutralizing and chemotactic effects on avian macrophages [83].

Immunoglobulins are vital elements of the humoral immune system, substantially enhancing immune responses such as phagocytosis and the elimination of pathogenic microorganisms [84]. *IgA* is one of the three primary immunoglobulin classes that respond to both localized and systemic illnesses [85]. Notably, when bacteria penetrate the gut epithelium, intestinal immunological cells initiate the production of cytokines, thereby augmenting the immune response against bacteria [86]. *S. Typhimurium* infection may enhance the transcription of *CCL20*, *TNF-α*, *IL-1β*, and *IL-6* genes, hence augmenting intestinal epithelial permeability [43]. Phytogenics possess anti-inflammatory and immune-modulating properties in diverse avian immunological and inflammatory conditions, showing beneficial effects on pro-inflammatory cytokines like *TNF-α*, *IL-6*, and *IL-1β*, thus alleviating gut inflammation and preserving its homeostasis [87]. Our results revealed that, in parallel with promoting blood and serum immunological metrics, dietary supplementation with PomNEs effectively augmented the reduced intestinal protective responses and reduced the elevated inflammatory response at 7 and 21 dpi with *S. Typhimurium* strain. At 7 and 21 days post-infection with *S. Typhimurium*, the incorporation of dietary PomNEs markedly reduced the mRNA transcriptional levels of genes associated with chemokines (*CCL20* and *CXCL13*) and pro-inflammatory cytokines (*TNF-α, IL-1β* and *IL-6*), as well as increasing the transcript levels of intestinal protective genes, including antimicrobial peptides (*AvBD6* and *AvBD12*) and *IgA*, thereby illustrating their substantial immune-boosting and anti-inflammatory properties. Accordingly, dietary pomegranate peel extract fortification markedly lessened the transcription levels of TNF-α and IL-6 genes, along with up-regulating the *IgA* gene in laying hens [88]. Dietary PPE inclusion significantly elevated IgM levels in laying hens [73]. Likewise, dietary PomNE supplementation significantly down-regulated the TNF-α and *IL-1β* genes in *E. coli*-challenged broilers and increased the IgA level in rabbits [89]. Consistent with our findings, dietary phytogenics markedly decreased the expression of *IL-1β* and *IL-6* genes in *S. Typhimurium*-challenged broilers [56]. Similarly, the incorporation of dietary phytogenics markedly down-regulated the *TNF-α*, *IL-1β*, *IL-6*, and *CCL20* genes, and up-regulated *AvBD6* and *AvBD12* genes in broilers; nonetheless, the effect of dietary PomNEs on genes related to chemokines, cytokines, antimicrobial peptides, and immunoglobulins in S. Typhimurium-challenged hens remains to be explored [90].

The avian antioxidant system is intricately connected to their overall wellness and immune condition, with gut antioxidant capacity serving as a critical indicator of their performance [91]. During the laying phase, the gastrointestinal epithelial cells of hens are especially vulnerable to infections and inflammation [92]. This vulnerability may result in impairments to barrier functions and immune equilibrium, intensify oxidative stress, and damage gut microbiota. As a result, these complications adversely impact bird metabolism and productive efficacy [93]. Bacterial infections in hens impair immune function, antioxidant status, and the production of ROS and nitrogen species, leading to physiological changes related to oxidative stress [94]. The overproduction of ROS can damage tissues, induce lipid peroxidation, and impair cellular activities, reducing bird performance, welfare, and survival, ultimately leading to financial losses [95]. The elimination of ROS via resilient endogenous antioxidant defense mechanisms, including GPX-1, SOD-1, and CAT enzymes, preserves the homeostasis of cells and protects against oxidative injury [96]. Moreover, oxidative stress can impair the transcription factors and redox-responsive signaling cascades, thereby undermining the cell’s biological activity. Through their enhancement of the synthesis of antioxidant proteins and phase-II detoxification enzymes, the transcription factors *NRF2*, *HO-1*, and *NQO1* are thought to have important regulatory roles in the response of cells to oxidative stress [97]. The principal mediators (*GPX*, *ROS*, and *COX-2*) mutually control the inflammation induced by bacterial infection. The enzyme *COX-2* mediates the conversion of arachidonic acid into inflammatory prostaglandins, thus initiating cytokine generation [98]. Consequently, administering natural feed supplements to poultry, especially phytogenics with immunostimulant properties, may augment their antioxidant reaction by neutralizing free radicals and alleviating the adverse impacts of ROS, thus enhancing meat and egg quality [99,100]. In this respect, PomNEs are natural antioxidants; nonetheless, further research is necessary to clarify their effects on the antioxidant potential of laying hens and to ascertain whether their administration could provide further benefits for strengthening this role. Our outcomes revealed a marked rise in the expression levels of *SOD-1*, *HO-1*, *GPX-1*, *CAT*, and *NQO1*, along with significant minimization of the expression of the *COX-2* gene in the intestinal and ovarian tissues of hens fortified with PomNEs, particularly PomNEs1.2, at 33 weeks of age (before *S. Typhimurium* infection at the conclusion of the peak laying phase). Dietary fortification with PomNEs, especially PomNEs1.2, alleviated the adverse effects of *S. Typhimurium* on the expression of antioxidant-related genes, restoring their levels close to those observed in the negative control group, proven by up-regulation of the decreased expression levels of intestinal and ovarian *SOD-1*, *CAT*, *HO-1*, *GPX-1*, and *NQO1* genes along with down-regulation of the elevated expression of intestinal and ovarian *COX2* at 45 weeks of age (conclusion of the mid-laying period) subsequent to *S. Typhimurium* infection. This suggests their function in activating the antioxidant reaction, thereby enhancing the hen’s immunity and general well-being. In accordance, recent investigations indicate that dietary pomegranate peel extract inclusion markedly elevated *SOD*, *CAT*, and *GPx* enzyme levels in laying hens [29,50,101]. Furthermore, previous research demonstrated that dietary PPE inclusion substantially up-regulated the transcript levels of *SOD, CAT*, and *GPX* genes in broilers, likely due to their polyphenolic constituents, which possess free-radical-scavenging and chelating characteristics [74]. In broilers, the free form of pomegranate peel has typically required gram-level dietary inclusion to exert measurable antioxidant effects; dietary pomegranate peel at 7.5–10 g/kg was needed to significantly upregulate hepatic catalase and *SOD1* and to lower circulating malondialdehyde in broiler chickens [74].

In the same vein, prior research revealed that dietary inclusion of phytogenic-loaded nano-emulsions markedly up-regulated *SOD-1*, *HO-1*, *GPX-1*, *CAT*, and *NQO1* genes, and markedly down-regulated the *COX-2* gene transcript level in broilers [102]. Pomegranate peel polyphenols are established activators of the Nrf2–Keap1 axis, and the coordinated up-regulation of the phase-II and antioxidant enzyme battery (*HO-1*, *NQO1*, *SOD-1*, *CAT*, and *GPX-1*) observed here is consistent with Nrf2-driven transactivation restoring enzymatic ROS scavenging and redox homeostasis in intestinal and ovarian tissue [103]. Because reactive oxygen species are upstream activators of redox-sensitive NF-κB signaling, this quenching of the oxidative burden provides a coherent explanation for the parallel suppression of the NF-κB-dependent pro-inflammatory module (*IL-1β*, *IL-6*, and *TNF-α*), *COX-2*, and the downstream chemokines *CCL20* and *CXCL13* [104]. Furthermore, *S. Typhimurium* exploits host intestinal inflammation to compete with the resident microbiota and expand in the gut, so by mitigating the inflammatory environment, PomNEs remove the ecological advantage upon which the pathogen depends [105]. At the same time, the up-regulation of avian β-defensins (*AvBD6* and *AvBD12*) and secretory *IgA* reinforces frontline innate and mucosal humoral defense. Moreover, polyphenols proven to mediate intestinal cell membrane destabilization, iron chelation, and interference with SPI-1 regulation reasonably underlie the dose-dependent down-regulation of the master regulator *hilA* and its effector *invA*, hence resulting in impaired epithelial invasion [106]. The convergence of restrained inflammation, reinforced barrier immunity, and direct anti-virulence action provides a unified explanation for the reduced *Salmonella* colonization of the ovaries, liver, and eggs, mechanistically linking the molecular findings to both pathogen clearance and the sustained productive performance.

Beyond the biological efficacy of PomNEs, the commercial production of pomegranate peel extract-loaded double nano-emulsions (PomNEs) rests on several practical points. First, pomegranate peel is produced in large quantities as an agricultural by-product. Second, the simple encapsulation process preserves storage stability, with the carboxymethyl cellulose double emulsion retaining phenolic content and antioxidant activity far better than the free extract, thereby extending the diet’s functional shelf life [22]. Third, dietary peel polyphenols are partly deposited in the egg, raising yolk phenolic content and lowering yolk malondialdehyde, a value-adding “functional egg” effect rather than a hazard, though residues of specific nano-delivered markers still warrant targeted quantification [19]. Finally, prolonged feeding appears safe: these biocompatible polyphenols have been fed across full production phases without adverse effects, and nano-delivery lowers the effective dose and anti-nutritional tannin risk [101]. The current research demonstrates that the inclusion of dietary PomNEs in challenged hens enhances their antioxidant competence. This enhancement may be due to the efficiency of nano-encapsulation in augmenting the bioavailability and bioactivity of PPE, which augments tissue penetration and absorption by cells, thus bolstering antioxidant reactions. Our evaluation indicates that dietary PomNEs possess immune-boosting, antibacterial, anti-inflammatory, and anti-virulence properties that mitigate the proliferation of *Salmonella* in laying hens. However, additional field validation is still required before their broader application can be recommended.

## 5. Conclusions

Under the experimental challenge conditions of this study, dietary supplementation with PomNEs improved laying performance in hens infected with an MDR *S. Typhimurium* strain. The most effective dose was the 1.2 g/kg level, accompanied by improvements in the hens’ antioxidant status, inflammatory response, and immune defense that may contribute to limiting *S. Typhimurium* colonization. These results suggest that PomNEs could represent a promising feed additive worth further evaluation as a potential complement or alternative to antimicrobial growth promoters, in line with broader efforts to reduce reliance on antimicrobials in poultry production. However, although the present findings demonstrate the efficacy of PomNEs under controlled experimental conditions, confirmation across other breeds, production phases, and *Salmonella* field isolates is required. In addition, longer-term, larger-scale studies, including cost–benefit assessments compared with the free form of pomegranate peel extract at the same selected doses, are needed before its practical application in large-scale farms.

## Figures and Tables

**Table 1:** Ingredients and nutrient composition of the experimental diets for laying hens (as dry matter).

Feed Stuffs (g/kg)	Pre-Laying (15–17 Weeks)	Laying (18–45 Weeks)
Yellow corn grain	62.25	61.10
Soybean meal, 47.5%	21.00	14.25
Calcium carbonate	5.00	7.50
Wheat bran	6.00	—
Dicalcium phosphate	1.65	2.50
Soybean oil	3.00	4.80
Corn gluten, 60%	—	8.75
Common salt	0.30	0.30
DL-Methionine, 98%	0.10	0.10
Lysine, HCl, 78%	0.10	0.30
Anti-mycotoxin	0.10	0.10
Premix *	0.50	0.30
Nutrient composition		
ME (kcal/kg) **	2956	3165
CF, %	2.87	2.09
EE, %	5.52	7.28
CP, %	16.47	17.14
Methionine, %	0.36	0.47
Lysine, %	0.91	0.95
Available P, %	0.42	0.55
Ca, %	2.42	3.57

* Vitamin–mineral premix supplied per kg diet: Vit. A, 12,000 IU; Vit. K3, 6.00 mg; Vit. E, 65 IU; Vit. B1, 3.40 mg; cyanocobalamin, 0.5 mg; Vit. B2, 6.65 mg; pyridoxine, 2.00 mg; pantothenic acid, 13.87 mg; Vit. D3, 2900 IU; Co, 0.25 mg; Se, 0.40 mg; Cu, 8.8 mg; I, 0.9 mg; Mn, 55 mg; Fe, 68.9 mg; Ca, 1.38 g; folic acid, 1.15 mg; biotin, 0.73 mg; niacin, 50 mg; and Zn, 76.8 mg. ** Computed according to [32]. ME, metabolizable energy; CP, crude protein; EE, ether extract; CF, crude fiber; Ca, calcium; P, phosphorus.

**Table 2:** Primer sequences used for the PCR procedures.

Target Gene	Primer Sequence (5′–3′)	Accession No./Reference
*Salmonella Typhimurium* virulence		
*invA*	F: GTGAAATTATCGCCACGTTCGGGCAA/R: TCATCGCACCGTCAAAGGAACC	KF026356
*hilA*	F: ATCGTCGGGAGTTTGCTATT/R: ACTGACCAGCCATGAAAAGA	[38]
Immune-related genes		
*CCL20*	F: AGGCAGCGAAGGAGCAC/R: GCAGAGAAGCCAAAATCAAAC	NM_204438
*TNF-α*	F: CCCCTACCCTGTCCCACAA/R: ACTGCGGAGGGTTCATTCC	XM_046900549.1
*IL-1β*	F: TTCCGGATGTATCTCGAGCA/R: GTGGATCGTGGTCGTCTTCA	NC_013670
*IL-6*	F: AGGACGAGATGTGCAAGAAGTTC/R: TTGGGCAGGTTGAGGTTGTT	NM_205498.1
*CXCL13*	F: GCCTGTGCCTGGTGCTC/R: TGCCCCCTTCCCCTAAC	NM_001348657.1
*IgA*	F: ACCACGGCTCTGACTGTACC/R: CGATGGTCTCCTTCACATCA	S40610.1
*AvBD12*	F: TGTAACCACGACAGGGGATTG/R: GGGAGTTGGTGACAGAGGTTT	NM_001001607.2
*AvBD6*	F: GCCCTACTTTTCCAGCCCTATT/R: GGCCCAGGAATGCAGACA	NM_001001193.1
Antioxidant genes		
*NQO1*	F: TCGCCGAGCAGAAGAAGATTGAAG/R: CGGTGGTGAGTGACAGCATGG	NM_001277620.1
*HO-1*	F: AAGAGCCAGGAGAACGGTCA/R: AAGAGCCAGGAGAACGGTCA	NM_205344
*COX-2*	F: TGTCCTTTCACTGCTTTCCAT/R: TTCCATTGCTGTGTTTGAGGT	NM_001167718.1
*CAT*	F: TGGCGGTAGGAGTCTGGTCT/R: GTCCCGTCCGTCAGCCATTT	NM_001031215.1
*SOD-1*	F: GGCAATGTGACTGCAAAGGG/R: CCCCTCTACCCAGGTCATCA	NM_205064.1
*GPX-1*	F: AACCAATTCGGGCACCAG/R: CCGTTCACCTCGCACTTCTC	HM590226
Housekeeping genes		
*GAPDH*	F: GGTGGTGCTAAGCGTGTTA/R: CCCTCCACAATGCCAA	NM205518
*rpoD*	F: ACATCGCTAAACGTATCGAA/R: GTACTGTTCCAGCAGATAGG	[39]

*invA*, *S. Typhimurium* invasion protein A gene; *hilA*, *S. Typhimurium* invasion protein regulator A gene; *IL*, interleukin; *CCL20*, chemokine C–C motif ligand 20; *TNF-α*, tumor necrosis factor α; *AvBD*, avian β-defensin; *IgA*, immunoglobulin A; *CXCL13*, stromal cell-derived factor 1; *COX-2*, cyclooxygenase 2; *rpoD*, RNA polymerase sigma factor; *GAPDH*, glyceraldehyde 3-phosphate dehydrogenase; *CAT*, catalase; *SOD-1*, superoxide dismutase 1; *GPX-1*, glutathione peroxidase 1; *HO-1*, haem oxygenase 1; *NQO1*, NAD(P)H quinone dehydrogenase 1.

**Table 3:** Impact of graded concentrations of dietary pomegranate peel extract-loaded nano-emulsions (PomNEs) on productive performance attributes in *Salmonella Typhimurium*-challenged Hy-Line Brown laying hens.

Parameter	Experimental Groups					*p*-Value	SEM
	NC	IC	PomNEs0.3	PomNEs0.6	PomNEs1.2		
Early laying period (18–22 weeks)							
HDEP %	55.89 ^b^	55.71 ^b^	55.94 ^b^	56.17 ^b^	60.34 ^a^	<0.001	0.70
Egg mass %	28.49 ^b^	28.34 ^b^	28.78 ^b^	29.03 ^b^	31.99 ^a^	0.043	0.24
Total FI (g/bird)	32,872	32,866	32,858	32,788	32,702	<0.001	0.86
FCR	3.29 ^a^	3.31 ^a^	3.26 ^a^	3.23 ^a^	2.87 ^b^	<0.001	0.004
NFEI	96.05 ^b^	95.67 ^b^	96.58 ^b^	97.93 ^b^	108.75 ^a^	<0.001	3.27
Peak laying period (23–33 weeks)							
HDEP %	96.10 ^b^	96.06 ^b^	96.20 ^b^	97.31 ^ab^	97.84 ^a^	0.002	0.25
Egg mass %	56.49 ^cd^	56.16 ^d^	57.72 ^bc^	58.58 ^b^	60.44 ^a^	<0.001	0.32
Total FI (g/bird)	11,741.88 ^a^	11,742.92 ^a^	11,495.40 ^b^	11,435.20 ^b^	11,291.00 ^c^	<0.001	1.43
FCR	3.92 ^a^	4.10 ^a^	3.51 ^b^	3.49 ^b^	3.28 ^c^	<0.001	0.001
NFEI	48.99 ^c^	48.69 ^c^	51.20 ^b^	52.27 ^b^	54.57 ^a^	<0.001	0.30
Terminal stage of mid-laying period (34–45 weeks)							
HDEP %	94.34 ^a^	56.76 ^d^	88.73 ^c^	91.59 ^b^	91.62 ^b^	<0.001	1.04
Egg mass %	60.62 ^a^	34.35 ^d^	54.81 ^c^	57.39 ^b^	57.92 ^b^	<0.001	0.43
Total FI (g/bird)	75,782 ^c^	78,320 ^a^	78,666 ^a^	76,846 ^b^	75,796 ^c^	<0.001	1.50
FCR	1.98 ^d^	3.62 ^a^	2.278 ^b^	2.13 ^c^	2.08 ^c^	<0.001	0.001
NFEI	70.66 ^c^	44.03 ^d^	75.78 ^b^	77.57 ^b^	81.33 ^a^	<0.001	0.74

HDEP %, hen-day egg production percentage; NFEI, net feed efficiency index; FCR, feed conversion ratio; FI, feed intake; NC, negative control; IC, infected control; PomNEs0.3/0.6/1.2, basal diet supplemented with PomNEs at 0.3, 0.6, and 1.2 g/kg, respectively. At 34 weeks of age, the IC and three treatment groups were infected with the MDR *S. Typhimurium* strain. Means within a row bearing different superscript letters (a–d) differ significantly (*p* < 0.05).

**Table 4:** Blood and serum immunological biomarkers of Hy-Line Brown laying hens in response to dietary PomNE fortification and *Salmonella Typhimurium* challenge.

Parameter	Experimental Groups					*p*-Value	SEM
	NC	IC	PomNEs0.3	PomNEs0.6	PomNEs1.2		
Before *Salmonella Typhimurium* challenge (33 weeks)							
Phagocytic %	59.80 ^d^	59.80 ^d^	63.80 ^c^	66.20 ^b^	70.40 ^a^	<0.001	0.96
Intracellular killing capacity %	70.09 ^d^	67.97 ^d^	73.79 ^c^	79.22 ^b^	83.39 ^a^	<0.001	1.09
LYZ (U/mL)	0.78 ^d^	0.64 ^e^	1.05 ^c^	1.46 ^b^	1.71 ^a^	<0.001	0.001
NO (µmol/L)	0.95 ^a^	1.04 ^a^	0.72 ^b^	0.61 ^c^	0.51 ^c^	<0.001	0.002
After *Salmonella Typhimurium* challenge (45 weeks)							
Phagocytic %	65.80 ^a^	53.20 ^b^	67.80 ^a^	67.00 ^a^	68.20 ^a^	<0.001	1.88
Intracellular killing capacity %	71.31 ^d^	62.91 ^e^	75.08 ^c^	80.49 ^b^	84.58 ^a^	<0.001	0.52
LYZ (U/mL)	0.91 ^d^	0.76 ^e^	1.18 ^c^	1.57 ^b^	1.81 ^a^	<0.001	0.001
NO (µmol/L)	0.56 ^d^	1.36 ^a^	0.78 ^b^	0.61 ^c^	0.53 ^d^	<0.001	0.001

LYZ, lysozyme; NO, nitric oxide. Group definitions as in Table 1. Means within a row bearing different superscript letters (a–e) differ significantly (*p* < 0.05).

## Data Availability

The original contributions presented in this study are included in the article/Supplementary Materials. Further inquiries can be directed to the corresponding authors.

## Supplementary Materials

The following supporting information can be downloaded at: https://www.mdpi.com/article/10.3390/vetsci13080775/s1, Detailed preparation and analysis of pomegranate peel extract Table S1: analysis of PPE and Table S2: Antimicrobial susceptibility profile of the field-isolated multidrug-resistant *Salmonella Typhimurium* strain used in the challenge trial with references [107,108,109].

## Abbreviations

Abbreviation	Definition
ANOVA	Analysis of variance
AOAC	Association of Official Analytical Chemists
ARRIVE	Animal Research: Reporting of In Vivo Experiments
*AvBD6*	Avian β-defensin 6
*AvBD12*	Avian β-defensin 12
BHI	Brain heart infusion
Ca	Calcium
*CAT*	Catalase
*CCL20*	Chemokine (C–C motif) ligand 20
CF	Crude fiber
CFU	Colony-forming unit
CLSI	Clinical and Laboratory Standards Institute
*COX-2*	Cyclooxygenase-2
CP	Crude protein
Ct	Threshold cycle
*CXCL13*	Chemokine (C–X–C motif) ligand 13/stromal cell-derived factor-1
DLS	Dynamic light scattering
DNA	Deoxyribonucleic acid
dpi	Days post-infection
EDTA	Ethylenediaminetetraacetic acid
EE	Ether extract
FCR	Feed conversion ratio
FI	Feed intake
*GAPDH*	Glyceraldehyde 3-phosphate dehydrogenase
GLM	General linear model
*GPX-1*	Glutathione peroxidase 1
HDEP	Hen-day egg production
*hilA*	*Salmonella* invasion protein regulator A gene
*HO-1*	Heme oxygenase-1
HPLC	High-performance liquid chromatography
IC	Infective control
ICK	Intracellular killing capacity
*IgA*	Immunoglobulin A
*IL-1β*	Interleukin-1 beta
*IL-6*	Interleukin-6
*invA*	*Salmonella* invasion protein A gene
ISO	International Organization for Standardization
LYZ	Lysozyme
MDR	Multidrug-resistant
ME	Metabolizable energy
mRNA	Messenger ribonucleic acid
NC	Negative control
NFEI	Net feed efficiency index
NO	Nitric oxide
*NQO1*	NAD(P)H quinone dehydrogenase 1
*NRF2*	Nuclear factor erythroid 2-related factor 2
P	Phosphorus
PBS	Phosphate-buffered saline
PCR	Polymerase chain reaction
PomNEs	Pomegranate peel extract-loaded nano-emulsion
PPE	Pomegranate peel extract
qPCR	Quantitative real-time polymerase chain reaction
ROS	Reactive oxygen species
*rpoD*	RNA polymerase sigma factor
RT-qPCR	Reverse transcription quantitative real-time polymerase chain reaction
SEM	Standard error of the mean
*SOD-1*	Superoxide dismutase 1
TEM	Transmission electron microscopy
*TNF-α*	Tumor necrosis factor alpha
wpi	Weeks post-infection
