# Supplementary material for: Enhanced Efficacy of Pomegranate Peel Extract via Double Nano-Emulsion Delivery in Laying Hens: Impact on Performance, Immunity, Antioxidant Status, and Salmonella Typhimurium Resistance
Source: Vet Sci. 2026 Aug 2;13(8):775. doi: 10.3390/vetsci13080775 (PMC13517583; doi:10.3390/vetsci13080775)
Supplement: Supplementary file 1 [file vetsci-13-00775-s001.zip › vetsci-4439652-supplementary.pdf]

Detailed preparation and analysis of pomegranate peel extract

Peels from pomegranates were carefully mashed, dried out at 50°C, after that extracted by 70% ethyl alcohol. The blend was then purified after being incubated at ambient temperature for 24 h at dark condition. After being dried out, the extracted material was lyophilized by freezing it for 24 h at -70°C. It was then stored at -20°C in a container that was shielded from light. The dried extracts were collected and weighed to calculate the percent of extract yield. The dried extracts were kept in dark at 4°c until used.

Percent of extract yield = Total dried extract yield per kg/ Kg pomegranate peel× 100

Total phenolic content

The total phenolic concentration of the extract was quantified by a colorimetric procedure based on the Folin–Ciocalteu reaction, as described by Li et al. (2006). The reaction absorbance was recorded spectrophotometrically against a gallic acid calibration series, and phenolic content was expressed as milligrams of gallic acid equivalents per 100 g of dry peel powder.

Total flavonoid content

Total flavonoids were measured using the aluminum chloride colorimetric assay following Li et al. (2006). Absorbance was read against a standard calibration curve, and results were expressed as milligrams of [quercetin / rutin] equivalents per 100 g of dry peel powder.

DPPH radical-scavenging activity

The capacity of the peel extract to donate hydrogen and neutralize free radicals was evaluated using a modified DPPH assay adapted from Singh et al. (2002). An

aliquot of 10 µL of the dried extract, dissolved in methanol at 0.01 g mL^{-1}, was combined with 1 mL of DPPH solution (500 µM) and brought to a final volume of 25 mL with methanol. The mixture was homogenized by vortexing and left to react in the dark at room temperature (25 ± 2 °C) for 20 min. At the end of the incubation, the fall in absorbance was recorded at 517 nm using a spectrophotometer. A control containing all reagents except the extract was run in parallel, and methanol served as the blank. Scavenging activity was expressed as percentage inhibition (I %) and calculated according to Lapornik et al. (2005):

$$I\ \% = [(A_C - A_S) / A_C] \times 100$$

where A_C is the absorbance of the control (all reagents without the test extract) and A_S is the absorbance in the presence of the extract.

HPLC analysis of pomegranate peel extract

The polyphenolic fractions of the PPE were measured using a high-performance liquid chromatography (HPLC) technique.

Chromatographic conditions

C18 column, 250 × 4.6 mm, 5 µm, held at 30 °C. Mobile phase A: water with 0.1% formic acid; B: acetonitrile (or methanol). A workable linear gradient: 5% B at 0 min → 30% B at 30 min → 60% B at 45 min → hold to 50 min → re-equilibrate 5–8 min. Flow 1.0 mL/min, injection 10–20 µL. DAD acquiring the full 200–600 nm spectrum, with quantification channels at 280 nm.

Identification and quantification

Confirm each peak by co-elution with its standard (spiking) plus UV spectral match. Quantify from the external calibration curves; report each compound as mg/100g dry peel, and record LOD, LOQ, R^2, and retention-time repeatability.

Table S1, analysis of PPE

Parameters	Pomegranate peel extract (PPE)
**Quantity of extract yield %**	23.12
**Total phenolics content**	614 mg/100gm powder
**Total flavonoids content**	8.35 mg/100gm powder
**Radical scavenging activity (Inhibition percent I %) at concentration 10µg/ml**	36.00
**Ellagic acid mg/100 g dry matter**	43.51
**Gallic acid mg/100 g dry matter**	56.43
**Punicalagin (α + β) mg/100 g dry matter**	619.74
**Punicalin mg/100 g dry matter**	52.66
**5-hydroxymethylfurfural mg/100 g dry matter**	11.29

Table S2. Antimicrobial susceptibility profile of the field-isolated multidrug-resistant *Salmonella* Typhimurium strain used in the challenge trial.

**Antimicrobial class**	**Antimicrobial agent**	**Interpretation**
Penicillins (aminopenicillins)	Amoxicillin	**R**
β-lactam / β-lactamase inhibitor combination	Amoxicillin–clavulanic acid	**R**
Cephalosporins (1st generation)	Cephalexin	**R**
Cephalosporins (3rd generation)	Cefotaxime	**R**
Cephalosporins (3rd generation)	Ceftazidime	**R**
Cephalosporins (3rd generation)	Ceftriaxone	**R**
Cephalosporins (4th generation)	Cefepime	**R**
Tetracyclines	Doxycycline	**R**
Tetracyclines	Oxytetracycline	**R**
Phenicols	Chloramphenicol	**R**
Macrolides	Erythromycin	**R**
Quinolones	Nalidixic acid	**R**
Polymyxins	Colistin	**R**
Folate pathway inhibitors	Sulfamethoxazole–trimethoprim	**R**

R: resistant.

References

Li, Y.; Guo, C.; Yang, J.; Wei, J.; Xu, J. and Cheng, S. (2006) Evaluation of antioxidant properties of pomegranate peel extract in comparison with pomegranate pulp extract. Food Chem; 2(96): 254-260.

Lapornik, B.; Prosek, M. and Wondra, A.G. (2005) Comparison of extracts prepared from plant by-products using different solvents and extraction time. J Food Eng; 2(71): 214-22.

Singh, R.P.; Chidambara-Murthy, K.N. and Jayaprakasha, G.K.(2002) Studies on the antioxidant activity of pomegranate (Punica granatum) peel and seed extract using in vitro models. J Agric Food Chem; 50(1): 81-6.
